# Two Antibody-Guided Lactic-co-Glycolic Acid-Polyethylenimine (LGA-PEI) Nanoparticle Delivery Systems for Therapeutic Nucleic Acids

**DOI:** 10.3390/ph14090841

**Published:** 2021-08-25

**Authors:** Jian-Ming Lü, Zhengdong Liang, Dongliang Liu, Bin Zhan, Qizhi Yao, Changyi Chen

**Affiliations:** 1 Michael E. DeBakey Department of Surgery, Baylor College of Medicine, One Plaza, Houston, TX 77030, USA; jianminl@bcm.edu (J.-M.L.); Zhengdong.Liang@uth.tmc.edu (Z.L.); donglial@bcm.edu (D.L.); qizhiyao@bcm.edu (Q.Y.); 2 National School of Tropical Medicine and Department of Pediatrics, Section of Tropical Medicine, Baylor College of Medicine, One Plaza, Houston, TX 77030, USA; bzhan@bcm.edu; 3 Center for Translational Research on Inflammatory Diseases (CTRID), Michael E. DeBakey VA Medical Center, Houston, TX 77030, USA

**Keywords:** polyethylenimine, LGA-PEI polymer, nanoparticle, active targeting delivery, therapeutic nucleic acid

## Abstract

We previously reported a new polymer, lactic-co-glycolic acid-polyethylenimine (LGA-PEI), as an improved nanoparticle (NP) delivery for therapeutic nucleic acids (TNAs). Here, we further developed two antibody (Ab)-conjugated LGA-PEI NP technologies for active-targeting delivery of TNAs. LGA-PEI was covalently conjugated with a single-chain variable fragment antibody (scFv) against mesothelin (MSLN), a biomarker for pancreatic cancer (PC), or a special Ab fragment crystallizable region-binding peptide (FcBP), which binds to any full Ab (IgG). TNAs used in the current study included tumor suppressor microRNA mimics (miR-198 and miR-520h) and non-coding RNA X-inactive specific transcript (XIST) fragments; green fluorescence protein gene (GFP plasmid DNA) was also used as an example of plasmid DNA. MSLN scFv-LGA-PEI NPs with TNAs significantly improved their binding and internalization in PC cells with high expression of MSLN in vitro and in vivo. Anti-epidermal growth factor receptor (EGFR) monoclonal Ab (Cetuximab) binding to FcBP-LGA-PEI showed active-targeting delivery of TNAs to EGFR-expressing PC cells.

## 1. Introduction

Cancer was the second leading cause of death, while the heart disease was the first leading cause of death in the United States (US) in 2018 [1]. However, cancer emerged recently as the leading cause of death in many states in the US [2]. It is estimated that the total new cancer cases and cancer deaths in US are 1,898,160 and 608,570, respectively, in 2021. New cases and death from pancreatic cancer (PC) in the US are estimated to be 60,430 and 48,220, respectively, in 2021. Now, PC is the third leading cause of cancer death following lung cancer (131,880 deaths) and colorectum cancer (52,980 deaths) [3]. For all stages combined, the 5-year relative survival rate is the lowest for PC (9%) among all cancer types. Even for patients with resectable disease, the overall survival rate is only 17% [3,4]. The poor prognosis is mainly due to lack of early diagnosis, tumor pathology, and progression and limited efficacy of available drugs and other therapies [3,5]. Therefore, there is an urgent need for the development of more effective therapeutic strategies against PC.

Therapeutic nucleic acid (TNA) is one of these new strategies for the treatment of many diseases, including cancer. TNA includes specific genes (DNA and mRNA), antisense oligonucleotides (ASOs), small interfering RNAs (siRNAs), microRNA (miRNA) analogs, miRNA inhibitors (antagonists), aptamers, ribozymes, CpG, decoy NAs, CRISPR-Cas, and other gene-editing tools. TNAs are used as tools, drugs, and vaccines for disease intervention or prevention at the genetic level. Unlike protein and small-molecule drugs, most TNAs specifically target mRNA or DNA gene (precursors of proteins) through Watson–Crick base pairing. Over the past few decades, TNAs have drawn great attention for research worldwide [6,7]; however, only a limited number of TNAs have been approved for clinical applications, such as Fomivirsen, Gendicine, Pegaptanib, Cambiogenplasmid, Alipogene tiparvovec, Mipomersen, Talimogene laherparepvec, Nusinersen, Eteplirsen, Strimvelis, Voretigene neparvovec, Patisiran, Inotersen, Onasemnogene abeparvovec, Givosiran, and COVID-19 vaccines (see Supplementary Table S1 for details). Currently, there are many clinical trials of TNAs, which are underway for different diseases [7,8]. However, the success of the TNAs has been limited due to the lack of safe and efficient delivery systems. Because of the poor bioavailability and instability in vivo, TNAs usually require delivery vectors or chemical modifications to help their stabilization and entering into the cell nucleus or cytosol to be effective. Compared with small molecule chemical drugs, naked DNA or RNA is prone to nuclease degradation; it is relatively large, negatively charged, and has difficulty passively crossing the cell membrane; it can activate the immune system and cause severe side effects [9,10]. For example, after intravenous administration of naked plasmid DNA in mice, its half-life in the blood is only a few min [11]. There are several barriers, which significantly limit the bioavailability of TNAs such as endothelial barriers, lysosome digestion, liver retention, renal clearance, and unexpected tissue accumulation [10].

The safe and effective delivery system for TNAs remains one of the most active research topics worldwide. In fact, many different types of delivery systems for TNAs have been developed including viral-based, non-viral-based, and combined hybrid systems for the treatment of a variety of diseases such as cancers, viral infections, hereditary diseases, genetic disorders, etc. Due to safety concerns regarding their immunogenicity and potential of integration, viral vectors such as adenoviruses, adeno-associated viruses (AAVs), retroviruses, and lentiviruses have limited clinical applications, while more and more non-viral-based systems (physical or chemical types) have been developed for broad clinical applications due to their safety profile and non-immunogenicity. Various chemicals such as natural or synthetic polymers, liposomes, dendrimers, cationic lipid materials, and peptides have been used for TNA delivery. Nanoscale non-viral vectors have aroused great interest because of their unique properties to facilitate TNA delivery.

Nanoparticles (NPs) are small in size, a few to a hundred nanometers (nm), but they have a relatively large surface area-to-volume ratio for efficiently carrying and absorbing other substances such as TNAs, thereby having a high TNA loading capacity. NP delivery systems can protect the enclosed TNAs from nuclease digestion and enhance cellular uptake, thus significantly increasing therapeutic efficacy. Especially for cancer therapy, NPs could be accumulated at tumor tissues through the mechanism of the enhanced vascular permeability and retention (EPR) effect as tumor tissues usually have leaky neovasculatures and lack effective lymphatic drainage [12,13].

Polyethylenimine (PEI) is one of the most widely investigated synthetic polymers for NP-based delivery of TNAs. Positively charged PEIs can readily condense negatively charged TNAs through electrostatic interactions to form compact and stable NPs [14,15]. PEIs exhibit a strong buffer capacity over a broad range of pH environments [16,17]. Positively charged amine groups of PEI can bind to negatively charged residues on the cell surface and get internalized into cells through clathrin-mediated endocytosis. PEI has a unique intracellular trafficking property that rescues TNAs from degradation in the endo-lysosomal compartment by inducing a proton sponge effect [18,19]. Amine groups in PEI inside the endosome can induce continuous proton pumping into the endosomes and reduce the pH, accompanying passive entry of chloride ions, and consequently excess influx of water, which results in the endosomes swelling and rupturing to release their contents, such as TNAs, into the cytoplasm [20]. Due to internal charge repulsion of PEI, the polymer itself is also able to expand (“umbrella” effect) when the protonation level increases, which contributes to the endosome rupturing as well [21]. In addition, PEI is able to increase nuclear-envelope permeability, which may help plasmid DNA entering into the nucleus [22]. Branched PEI (such as 25 kDa) is one of the most commonly used TNA delivery vectors in vitro and in vivo due to its highly effective endosomal escape activity. However, clinical use of branched PEI has been limited because of its potential toxicity caused by the disruption of the cell membrane or damage of the mitochondrial membrane at relatively high doses [23,24]. The toxicity of PEI has been associated with its molecular weight and structure. Free PEI seems more toxic than the PEI-NA complex [25].

In order to reduce the potential toxicity of branched PEI for TNA delivery, chemical modifications of PEI have been actively studied. In fact, rational designs of chemical modifications of branched PEI or conjugation of targeting groups or PEGylation are relatively easy because of the presence of many amino groups in PEI [26]. For examples, PEI has been modified by various polymers, hydrophobic chemicals, and other molecules such as dextran, cyclodextrins, chitosan, polyethylene glycol, polyethylene glycol (PEG), acetate, butanoate, hexanoate, folic acid, peptides, proteins, metals, and more. PEI-based delivery systems for TNAs have been broadly investigated in clinical trials for HIV vaccines and many types of cancers, including PC [27]. Recently, we developed a new modified PEI, in which lactic-co-glycolic acid (LGA) single units are covalently linked to the branched PEI (25 KDa). The new LGA-PEI polymer spontaneously formed NPs with TNAs and showed much lower toxicity than control PEI NPs in mouse models and in cell cultures, while also showing high transfection efficiency for DNA or RNA in vitro and in vivo [28].

One of the important strategies of PEI modification is to achieve an active-targeting delivery of TNAs, improving therapeutic efficacy while minimizing off-target damage in healthy tissues [29]. Antibodies (Abs) or Ab fragments as targeting ligands are the most commonly used for conjugation on the surface of NPs for active-targeting delivery because they have high affinity to specifically bind to antigens or receptors on cancer cell surfaces [30]. In addition, various types of proteins, small peptides, or other molecules were used to conjugate on the surface of NPs to achieve active targeting to cancer cells, including transferrin, arginine-ycine-aspartic acid (RGD) peptide, cyclic arginine-glycine-aspartic acid-tyrosine-lysine c(RGDyK), bombesin peptide, NR7 peptide, hyaluronic acid (HA), folate, aptamers, galactose, and lactose [30]. Modifying the PEIs using targeting ligands specifically bind unique cell surface markers of diseased cells, not expressed on the normal healthy cells. For example, humanized anti-Her-2/neu monoclonal Ab, Trastuzumab, covalently conjugated to branched PEI (25 KDa), more effectively delivered plasmid DNA into Her-2/neu-expressing breast cancer cells [31]. We and others have reported that mesothelin (MSLN) is widely expressed in human cancers; for example, the majority (80–90%) of PC and ovarian cancers and 100% of epithelial mesotheliomas highly express MSLN [32,33,34,35,36,37,38,39]. It is also overexpressed in lung adenocarcinomas, gastric cancers, triple-negative breast cancers, uterine serous carcinoma, acute myeloid leukemia, and cholangiocarcinoma [40,41,42,43,44,45,46,47,48,49,50,51] (Supplementary Table S2). However, MSLN has only limited expression in mesothelial cells lining the pleura, pericardium, and peritoneum [39]; no MSLN expression is found in solid organs such as the heart, kidney, and liver [34]. Thus, MSLN has become a unique biomarker for targeting cancer therapy. In the current study, we successfully covalently linked a single-chain variable fragment antibody against MSLN (MSLN scFv) to our LGA-PEI polymer and showed MSLN-targeted delivery of TNAs in PC cells and animal models. Furthermore, we rationally designed and conjugated our LAG-PEI polymer with a special Ab fragment crystallizable region (Fc)-binding peptide (FcBP) [52], which is able to bind to IgG (full Abs) for active-targeting delivery. Since the expression of MSLN is closely associated with the high expression of cell surface epidermal growth-factor receptor (EGFR) in PC cells [53], we used a clinical grade anti-EGFR Ab (Cetuximab, C255) for its binding to FcBP-LGA-PEI NPs, achieving targeting delivery of TNAs in PC cells with high expression of EGFR. EGFR is an important molecular marker and target for many types of cancers including PC, breast cancer, glioblastomas, and colorectal cancer [54,55,56,57]. In the current study, we developed two targeting-delivery systems based on our new LGA-PEI polymer, which may deliver a variety of types of potential TNAs including DNA, miRNA, and modified mRNA, and may have broad clinical applications for effective treatments of PC and other types of cancers that express specific biomarkers.

We tested the delivery of miR-198 mimics, miR-520h mimics, and non-coding RNA X-inactive specific transcript (XIST) fragments, which may have therapeutic potential for cancers. We previously identified a novel network “interactome” of tumorigenic prognostic factors including miR-198, MSLN, NF-kB, homeobox transcription factor POU2F2 (OCT-2), pre-B cell leukemia homeobox factor 1 (PBX-1), and valosincontaining protein (VCP). More importantly, this interactome is interconnected through a tumor suppressor, miR-198, which is able to both directly and indirectly modulate the expression of various factors of this network and plays a critical role in PC pathogenesis [58]. When miR-198 expression is reduced in PC tissues, patient survival is dismal; while miR-198 level is relatively high, patients have a better prognosis and increased survival. In addition, miR-198 replacement reverses tumorigenicity in experimental models. These data indicate that miR-198 has therapeutic potential for PC. miR-198 has been shown to have a potent tumor-suppressor function in other types of cancers including renal cell carcinoma, ovarian cancer, lung cancer, and prostate cancer [59,60,61,62]. Furthermore, many studies have demonstrated that miR-520h could significantly inhibit tumor progression or prevent chemotherapy drug resistance in different types of cancers such as PC, multiple myeloma, and gastric cancer [63,64,65]. It has been reported that XIST plays a tumor-suppressor function in many types of cancers including classical Hodgkin’s disease, renal cell carcinoma, breast cancer, and prostate cancer [66,67,68,69]. Green fluorescence protein gene (GFP) was also used as an example of plasmid DNA. In the current study, we focused on the production and characterization of our new delivery systems as well as their delivery efficiency of these potential TNAs in vitro and in vivo.

## 2. Results

### 2.1. MSLN scFv-LGA-PEI and Anti-EGFR Ab/FcBP-LGA-PEI Polymers Effectively Load NAs and Form Functional NPs

**Figure 1:**
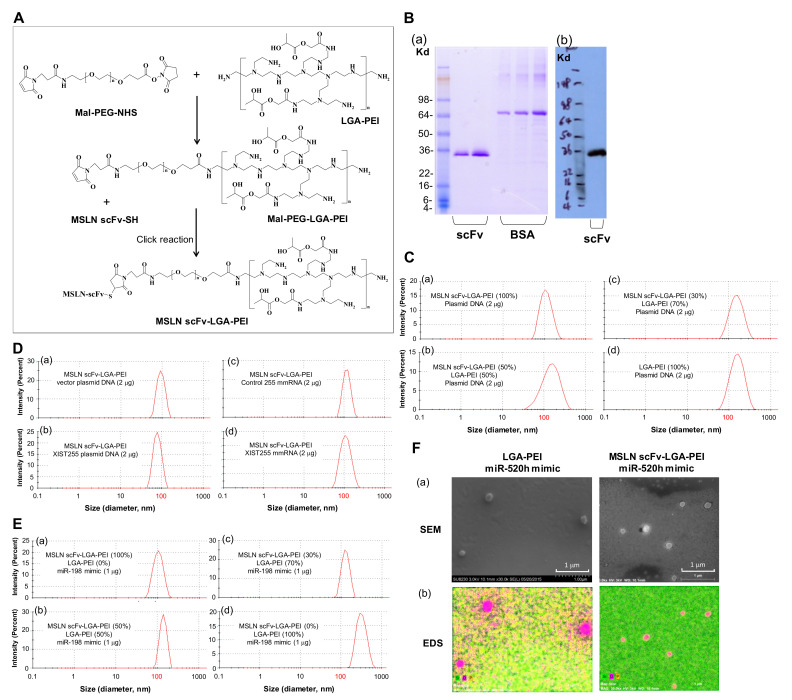
Synthesis of MSLN scFv-LGA-PEI and nanoparticle (NP) formation with different types of nucleic acids. (**A**) Proposed mechanism for the conjugation of MSLN scFv to LGA-modified PEI polymers. Bi-functional PEG (Mal-PEG-NHS) was used to covalently link to the MSLN scFv fragment through a click reaction. (**B**) Recombinant MSLN scFv was produced from the yeast expression system. (**a**) SDS Page and Coomassie blue staining showing MSLN scFv. (**b**) Western blot (anti-His Ab) showing the molecular weight of MSLN scFv (about 30 kDa). (**C**) Effect of different MSLN scFv-LGA-PEI and LGA-PEI ratios on the size of NP formation with plasmid DNA (**a**–**d**). Average sizes are listed in Supplementary Table S3. (**D**) NP sizes formed from of pure MSLN scFv-LGA-PEI (5 μg) with 2 μg vector plasmid (double-stranded, circular DNA, 3.2 kbp) (**a**), XIST255 gene containing plasmid DNA (**b**), XIST255 mmRNA (single-stranded RNA, 255 bases) (**c**) or control mmRNA (**d**). (**E**) The size distribution of NP formation from two polymers, MSLN scFv-LGA-PEI and LGA-PEI at different ratios (**a**–**d**) with miR-198 mimics (double-stranded, 23 bp). Average sizes are listed in Supplementary Table S4. (**F**) Scanning electronic microscopy (SEM) and energy-dispersive spectroscopy (EDS) analysis of LGA-PEI/miR-520h mimics NPs, and MSLN scFv-LGA-PEI/miR-520h mimics. (**a**) SEM images. (**b**) EDS composition map (oxygen element in purple and phosphorus element in yellow color).

We previously reported that synthesized LGA-PEI polymers still have about 50% primary amine groups of PEI left [28]. In the current study, we established two Ab-guided LGA-PEI NP delivery systems for TNAs. The first is MSLN scFv-LGA-PEI polymer, for which MSLN scFv is covalently linked to LGA-PEI, and the second is FcBP-LGA-PEI polymer, in which FcBP is covalently linked to LGA-PEI; any full Ab (IgG) can bind to the FcBP-LGA-PEI polymer. For the preparation of the MSLN scFv-LGA-PEI polymer (Figure 1A and Figure S1), we used bi-functional PEG (Mal-PEG-NHS) to covalently link the MSLN scFv fragment to LGA-PEI. First, Mal-PEG-NHS reacted with LGA-PEI at a desired molar ratio in 0.1 M phosphate buffer (pH 7.0) at room temperature for 3 h. Primary amine of the PEI attacked the ester bond of Mal-PEG-NHS, thus replacing the NHS group to conjugate Mal-PEG to PEI of the LGA-PEI through amide linkage. The Mal-PEG-PEI-LGA was purified by gel-permeation chromatography. The MSLN scFv fragment was then added to the Mal-PEG-PEI-LGA at a desired molar ratio and reacted at room temperature overnight under nitrogen. The hydrosulfyl group (-SH) (thiol) of cysteine in the peptide could add to the C=C double bond of the maleimide group in Mal-PEG-PEI-LGA, forming a stable S-C bond (click reaction). The yield of MSLN scFv-LGA-PEI and FcBP-LGA-PEI was about 70%, estimated from the starting material and final product weights. Therefore, the MSLN scFv fragment was conjugated with LGA-PEI. We produced a recombinant MSLN scFv (SS1) based on the published gene sequence identified by phage display [70,71]. We cloned and expressed MSLN scFv as a soluble recombinant protein with a His tag in yeast (*Pichia pastoris* X33 strain). SDS-PAGE assay indicated that MSLN scFv is a 30 kDa protein with 95% purity (Figure 1B). The MSLN scFv-LGA-PEI was purified by dialysis against 0.1 M NaCl. This MSLN scFv-LGA-PEI polymer is ready for loading TNAs and specifically delivering to cancer cells with the expression of MSLN. MSLN scFv-LGA-PEI polymers effectively load different types of NAs including plasmid DNA, microRNA (miRNA) mimics, and modified mRNAs (mmRNAs). For example, NPs were formed from a total of 5 μg of two polymers, MSLN scFv-LGA-PEI and LGA-PEI, at different ratios with 2 μg of plasmid DNA (double-stranded, circular DNA, 4.7 kbp) in 50 μL water. The NP solution was diluted 100 times in water and the particle size was measured by the dynamic light-scattering method (Zetasizer Nano ZS90, Malvern, Worcestershire, UK). The average particle sizes varied slightly with the ratios of the MSLN scFv-LGA-PEI and LGA-PEI polymers, ranging from 112 nm at 100% MSLN scFv-LGA-PEI with DNA to 153 nm at 100% LGA-PEI with DNA (Figure 1C and Supplementary Table S3). Additionally, NPs were formed from pure MSLN scFv-LGA-PEI (5 μg) with 2 μg vector plasmid (double-stranded, circular DNA, 3.2 kbp) and plasmid DNA containing XIST gene fragments (255 nucleotides), XIST255 mmRNA (single stranded RNA, 255 bases), or control mmRNA. Average particle sizes were around 100 nm. NPs formed from pure MSLN scFv-LGA-PEI and DNA showed smaller particles sizes than those formed from MSLN scFv-LGA-PEI mixed with LGA-PEI and DNA or pure LGA-PEI and DNA (Figure 1D). Furthermore, NPs were also formed from a total of 1.5 μg of two polymers, MSLN scFv-LGA-PEI and LGA-PEI, at different ratios with 1 μg of miR-198 mimics (double-stranded, 23 bp) in 50 μL water. The average particle sizes varied slightly with the ratios of MSLN scFv-LGA-PEI and LGA-PEI polymers, ranging from 126 nm at 100% MSLN scFv-LGA-PEI with miR-198 mimics to 332 nm at 100% LGA-PEI with miR-198 mimics (Figure 1E and Supplementary Table S4). As an example, scanning electronic microscopy (SEM) and energy-dispersive spectroscopy (EDS) analysis confirmed NP formation from LGA-PEI (1.5 μg)/miR-520h mimics (1 μg), and from MSLN scFv-LGA-PEI (1.5 μg)/miR-520h mimics (1 μg). The SEM images indicated that the particle sizes were about 100–200 nm; the EDS analysis showed these particles containing a large amount of oxygen and phosphorus elements, indicating particles containing NAs (Figure 1F).

**Figure 2:**
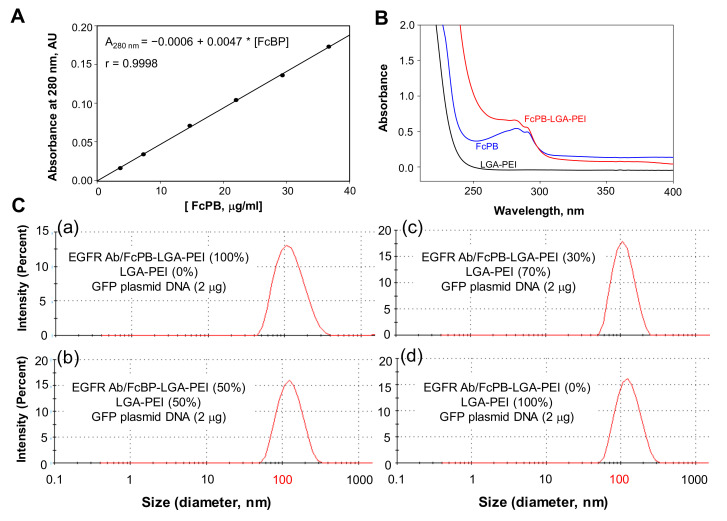
Synthesis of the FcBP-LGA-PEI polymer and nanoparticle (NP) formation with whole antibody (Ab) and nucleic acids. Conjugation of FcBP with the LGA-PEI polymer for binding full Ab for the targeting delivery. PEG (Mal-PEG-NHS) was covalently linked to LGA-PEI (Supplementary Figure S1). FcBP was covalently linked to the Mal-PEG-PEI-LGA though a click reaction. (**A**) Standard curve of spectroscopic analysis for the FcBP standard at 280 nm with different concentrations. Where f = y0 + a ∗ x, y0 = −0.0006, a = 0.0047, R = 0.9998. The solvent was phosphate buffer (pH 7.2). (**B**) Confirmation of conjugation of FcBP to the LGA-PEI polymer, and determination of FcBP content in the FcBP-LGA-PEI polymer. The concentration of FcBP was 65 μg/mL, while LGA-PEI was 10 mg/mL. (**C**) Effect of different anti-EGFR Ab/FcBP-LGA-PEI and LGA-PEI ratios on the size of NP formation with plasmid DNA. Full Ab (anti-EGFR Ab) is docked into FcBP-LGA-PEI to form the anti-EGFR Ab/FcBP-LGA-PEI polymer. NPs are formed from a total of 5 μg of two polymers, anti-EGFR Ab/FcBP-LGA-PEI and LGA-PEI at different ratios with 2 μg plasmid DNA (double-stranded, circular DNA, 4.7 kbp) in 100 μL DI water (**a**–**d**). NP sizes are measured with dynamic light scattering (Zetasizer Nano ZS90). Average sizes are listed in Supplementary Table S5.

For preparation of the FcBP-LGA-PEI polymer, we covalently conjugated the FcBP (DCAWHLGELVWCT), previously discovered by bacteriophage display and which shared the same binding site of the Fc region of IgG Abs from bacteria *Staphylococcus aureuss* protein A and *treptococcal* protein G [52], to LGA-PEI through a link molecule (bi-functional PEG, Mal-PEG-NHS). Extra glycine residues are added to both sides of the FcBP to enhance their conformational flexibility and to provide full access for the Ab. Cysteine is located at both ends of the FcBP, CGGGGDCAWHLGELVWCTGGGGC. The cysteine in one end is used to conjugate the peptide to LGA-PEI through bi-functional PEG, Mal-PEG-NHS. We used bi-functional PEG (Mal-PEG-NHS) to connect FcBP to LGA-PEI. Mal-PEG-NHS reacted with LGA-PEI to form Mal-PEG-PEI-LGA. The FcBP was added to the Mal-PEG-PEI-LGA at a desired molar ratio though a click reaction between the thiol of cysteine in the peptide and maleimide group in Mal-PEG-PEI-LGA, forming a stable S-C bond. Therefore, FcBP was conjugated with LGA-PEI (Supplementary Figure S1). The FcBP-PEG-PEI-LGA was further purified by dialysis against 0.1 M NaCl. This new FcBP-LGA-PEI polymer was used for loading specific Abs to the NP for active-targeting delivery to specific cell types. The FcBP has an absorbance at 280 nm, while LGA-PEI does not. The standard curve of spectroscopic analysis for the FcBP standard at 280 nm with different concentration was established to detect FcBP content covalently linking to LGA-PEI (Figure 2A). Successful FcBP conjugation into LGA-PEI was confirmed by spectrophotometry analysis (Figure 2B). The new polymer FcBP-LGA-PEI also had absorbance at 280 nm, which showed a similar peak as that of FcBP, indicating the conjugation of FcBP to the LGA-PEI polymer. For FcBP-LGA-PEI (10 mg/mL), A280 is about 0.32 after corrected from baseline and background subtraction; so [FcBP] = (0.32 + 0.0006)/0.0047 = 68 μg/mL, and thus, 100% × 0.068(μg/mL)/10 (mg/mL) = 0.68%. By using FcBP as a standard, we estimate that the FcBP-LGA-PEI contains about 0.68% FcBP in weight. The new FcBP-LGA-PEI polymer effectively loaded plasmid DNA and included a full Ab, anti-EGFR Ab, as an example, to form NPs, which were confirmed by dynamic light-scattering analysis (Figure 2C and Supplementary Table S5). Anti-EGFR Ab attached to the FcBP-LGA-PEI/DNA NPs did not increase the particle sizes based on the dynamic light-scattering measurements. Nucleic acid loading efficiency of our polymers (LGA-PEI, MSLN scFv-LGA-PEI and anti-EGFR Ab/FcBP-LGA-PEI) was confirmed by gel retardation assay (Supplementary Figure S2).

### 2.2. MSLN scFv-LGA-PEI and Anti-EGFR Ab/FcBP-LGA-PEI NPs with NAs Specifically Enhance Their Binding and Internalization into Targeting PC Cell Lines

**Figure 3:**
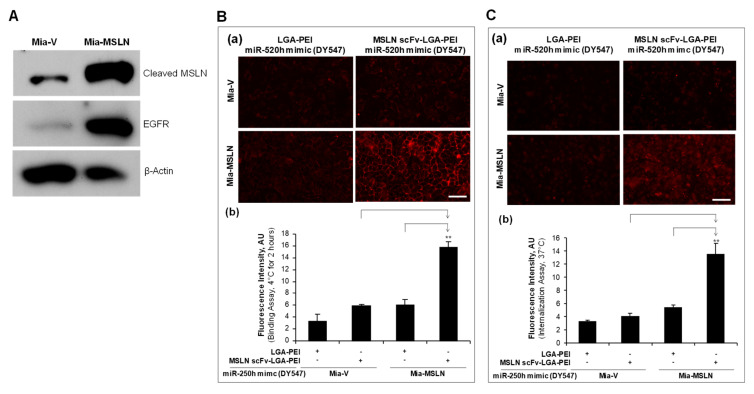
Targeting delivery of MSLN scFv-LGA-PEI nanoparticles (NPs) with miR-520h mimics to specific cells in vitro. (**A**) Stable transfection of mesothelin (MSLN) in pancreatic cancer cell line Mia-CaPa-2 cells (Mia-MSLN) also showed high expression of EGFR (Western blot analysis). (**B**) Cell-binding assay of MSLN scFv-LGA-PEI/miR-520h mimics NPs. miR-520h mimics were labeled with a fluorescence dye DY547 and loaded into either LGA-PEI or MSLN scFv-LGA-PEI polymer to form NPs, which were incubated with Mia-MSLN and Mia-vector control cells (60–70%) at 4 °C for 2 h before taking images. (**a**) MSLN scFv-LGA-PEI/miR-520h mimics NPs enhance binding to MSLN high-expressing cells (Mia-MSLN). (**b**) Fluorescence intensity was measured from images in each group (*n* = 5). The MSLN scFv-LGA-PEI/miR-520h mimics NPs showed a highest binding to the MSLN overexpressed cells. ** *p* < 0.01. (**C**) Cell internalization assay of MSLN scFv-LGA-PEI/miR-520h mimics NPs. MSLN scFv-LGA-PEI/miR-520h mimics or LGA-PEI/miR-520h mimics NPs were incubated with Mia-MSLN and Mia-vector control cells at 4 °C for 2 h, and then the cells were replaced with fresh culture medium and cultured at 37 °C overnight before taking images. (**a**) MSLN scFv-LGA-PEI/miR-520h mimics showed enhanced internalization to Mia-MSLN cells. (**b**) Fluorescence intensity was measured from images in each group (*n* = 5). Mia-MSLN cells treated with MSLN scFv-LGA-PEI/miR-520h mimics NPs showed the strongest fluorescence, indicating MSLN mediated the strong internalization of the NPs. ** *p* < 0.01. Scale bar (50 µm).

**Figure 4:**
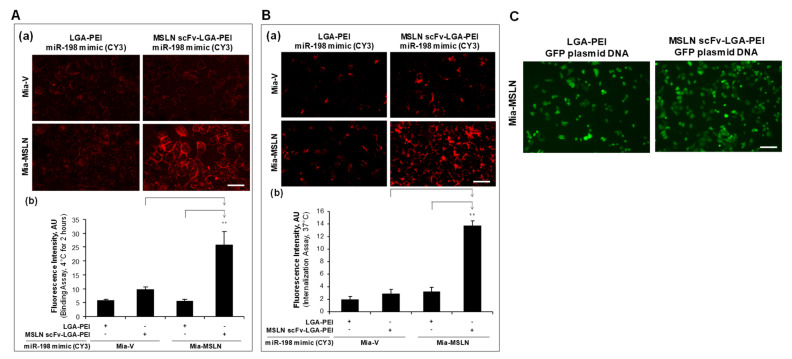
Targeting delivery of MSLN scFv-LGA-PEI nanoparticles (NPs) with/miR-198 mimics to specific cells in vitro. (**A**) Cell binding assay of MSLN scFv-LGA-PEI/miR-198 mimics NPs. miR-198 mimics were labeled with a fluorescence dye Cy3 and loaded into either LGA-PEI or MSLN scFv-LGA-PEI polymer to form NPs, and were incubated with Mia-MSLN and Mia-vector control cells at 4 °C for 2 h before taking images. (**a**) MSLN scFv-LGA-PEI/miR-198 mimics NPs showed enhanced binding to Mia-MSLN cells. (**b**) Fluorescence intensity was measured from images in each group (*n* = 5). Mia-MSLN cells treated with MSLN scFv-LGA-PEI/miR-198 mimics NPs showed the strongest fluorescence, indicating a strong binding to the MSLN overexpressed cells. ** *p* < 0.01. (**B**) Cell internalization assay of MSLN scFv-LGA-PEI/miR-198 mimics NPs. MSLN scFv-LGA-PEI/miR-198 mimics NPs or LGA-PEI/miR-198 mimics NPs were incubated with Mia-MSLN and Mia-vector control cells at 4 °C for 2 h; then, the cells were replaced with fresh culture medium and cultured at 37 °C overnight before taking images. (**a**) MSLN scFv-LGA-PEI/miR-198 mimics NPs showed enhanced internalization to MSLN high-expressing cells (Mia-MSLN). (**b**) Fluorescence intensity was measured from images in each group (*n* = 5). Mia-MSLN cells treated with MSLN scFv-LGA-PEI/miR-198 mimics NPs showed the strongest fluorescence, indicating MSLN mediated the strong internalization of the NPs. ** *p* < 0.01. (**C**) Cell transfection of MSLN scFv-LGA-PEI/GFP plasmid DNA NPs in Mia-MSLN cells. MSLN scFv-LGA-PEI/GFP plasmid DNA NPs NPs were prepared (details in the Materials and Methods section). Cells were incubated with NPs for 24 h before taking fluorescence images. Gene expression was measured by observing GFP-positive cells under a fluorescent microscope. Cells treated with MSLN scFv-LGA-PEI/GFP plasmid DNA NPs showed a stronger green fluorescence than cells treated with LGA-PEI/GFP plasmid DNA NPs, indicating MSLN-mediated GFP expression. Scale bar (50 µm).

In our previous study [32], we established stable transfection of MSLN in PC cell line Mia-CaPa2 (Mia-MSLN cells) as well as vector control cells (Mia-vector cells). In the current study, we confirmed that Mia-MSLN cells have a higher expression of MSLN than Mia-vector cells. Furthermore, EGFR expression in Mia-MSLN cells was much higher than that in Mia-vector cells (Figure 3A). MSLN could enhance EGFR expression in PC cells [53]. Both MSLN and EGFR molecules were used as targets for our Ab-guided NP delivery systems. For the MSLN scFv-LGA-PEI system, we loaded the MSLN scFv-LGA-PEI/LGA-PEI polymer or LGA-PEI polymer (control) with fluorescence labelled miR-520h mimics or miR-198 mimics to form NPs, and determined their binding and internalization efficiency to Mia-MSLN cells as compared with Mia-vector cells. Indeed, MSLN scFv-LGA-PEI/miR-520h mimics NPs (Figure 3B,C) or MSLN scFv-LGA-PEI/miR-198 mimics NPs (Figure 4A,B) showed a significantly higher level of both cell binding and internalization in Mia-MSLN cells than those in Mia-vector cells. To further confirm the gene delivery efficiency, MSLN scFv-LGA-PEI polymer or LGA-PEI polymer as a control was loaded with green fluorescence protein (GFP)-gene-containing plasmid to form NPs and transfected to Mia-MSLN cells for 24 h. GFP expression was much higher with the delivery of MSLN scFv-LGA-PEI/GFP plasmid NPs than that of LGA-PEI/GFP plasmid NPs, indicating the effect of MSLN scFv targeting MSLN on Mia-MSLN cells (Figure 4C).

**Figure 5:**
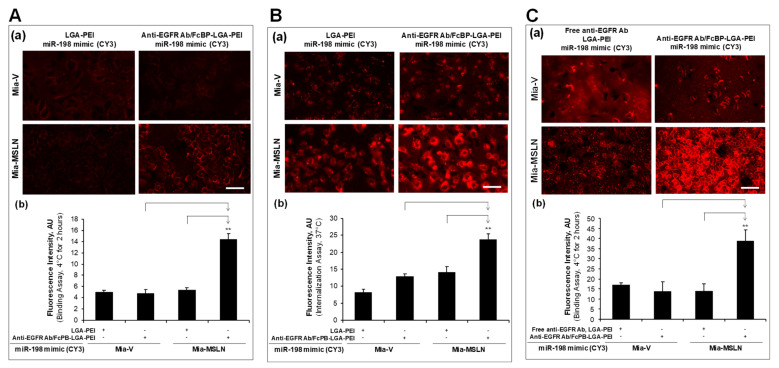
Targeting delivery of anti-EGFR Ab/FcBP-LGA-PEI nanoparticles (NPs) with nucleic acids to specific cells in vitro. (**A**) Cell binding assay of anti-EGFR Ab/FcBP-LGA-PEI/miR-198 mimics NPs and LGA-PEI/miR-198 mimics NPs. Fluorescence (Cy3)-labeled anti-EGFR Ab/FcBP-LGA-PEI/miR-198 mimics NPs or LGA-PEI/miR-198 mimics control NPs were added to cell culture of Mia-MSLN cells and Mia-vector control cells at 70% confluence at 4 °C for 2 h before taking images. (**a**) anti-EGFR Ab/FcBP-LGA-PEI/miR-198 mimics NPs showed enhanced binding to Mia-MSLN cells. (**b**) Fluorescence intensity was measured from images in each group (*n* = 5). ** *p* < 0.01. (**B**) Cell internalization assay of anti-EGFR Ab/FcBP-LGA-PEI/miR-198 mimics NPs and LGA-PEI/miR-198 mimics NPs. Anti-EGFR Ab/FcBP-LGA-PEI/miR-198 mimics NPs or LGA-PEI/miR-198 mimics NPs were added into cell cultures of Mia-MSLN and Mia-vector control cells at 4 °C for 2 h; then, the cells were replaced with fresh culture medium and cultured at 37 °C overnight before taking images. (**a**) Anti-EGFR Ab/FcBP-LGA-PEI/miR-198 mimics NPs showed enhanced internalization in Mia-MSLN cells. (**b**) Fluorescence intensity was measured from images in each group (*n* = 5). ** *p* < 0.01. (**C**) Cell binding assay of anti-EGFR Ab/FcBP-LGA-PEI/miR-198 mimics NPs and free anti-EGFR Ab with LGA-PEI/miR-198 mimics NPs. miR-198 mimics (1 μg) labeled by a fluorescence dye Cy3 were loaded to either free anti-EGFR Ab with LGA-PEI (1.5 μg) or anti-EGFR Ab/FcBP-LGA-PEI (1.5 μg) to form NPs, which were added to the cell cultures of Mia-MSLN and Mia-vector cells at 70% confluence at 4 °C for 2 h before taking images. (**a**) anti-EGFR Ab/FcBP-LGA-PEI/miR-198 mimics NPs showed enhanced binding to Mia-MSLN cells. (**b**) Fluorescence intensity was measured from images in each group (*n* = 5). ** *p* < 0.01. Scale bar (50 µm).

**Figure 6:**
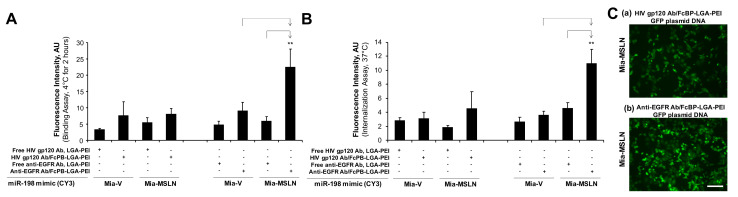
Comparison of specific antibody and non-specific antibody for the targeting delivery of FcBP-LGA-PEI nanoparticles (NPs) with miR-198 mimics to specific cells in vitro. (**A**) Cell-binding assay of FcBP-LGA-PEI/miR-198 mimics NPs with different Abs. Mia-MSLN or Mia-vector cells were treated with FcBP-LGA-PEI/miR-198 mimics NPs with anti-EGFR Ab or HIV gp120 Ab. For controls, Mia-MSLN or Mia-vector cells were treated with LGA-PEI/miR-198 mimics with free anti-EGFR Ab or HIV gp120 Ab. All cells were cultured at 4 °C for 2 h before taking images and performing fluorescence intensity analysis (*n* = 5). ** *p* < 0.01. (**B**) Cell internalization assay of FcBP-LGA-PEI/miR-198 mimics NPs with different Abs. Mia-MSLN or Mia-vector cells were treated with FcBP-LGA-PEI/miR-198 mimics NPs with anti-EGFR Ab or HIV gp120 Ab. For controls, Mia-MSLN or Mia-vector cells were treated with LGA-PEI/miR-198 mimics with free anti-EGFR Ab or HIV gp120 Ab. All cells were cultured at 4 °C for 2 h; then, the cells were replaced with fresh culture medium and cultured at 37 °C overnight before taking images and performing fluorescence intensity analysis. ** *p* < 0.01. (**C**) Targeting delivery of GFP plasmid DNA into Mia-MSLN cells. Anti-EGFR Ab/FcBP-LGA-PEI/GFP plasmid DNA NPs were prepared (details in the Section 4). The NPs were added to 10–15 k Mia-MSLN cells/well growing in 24-well plates and cultured for 24 h. Gene expression was measured by observing GFP-positive cells under a fluorescent microscope. Scale bar (50 µm).

For the anti-EGFR Ab/FcBP-LGA-PEI system, we loaded the FcBP-LGA-PEI polymer or the LGA-PEI polymer with fluorescence-labelled miR-198 mimics to form NPs; anti-EGFR Ab was bound to FcBP-LGA-PEI/miR-198 mimics to form the final anti-EGFR Ab/FcBP-LGA-PEI/miR-198 mimics NPs. Both Mia-MSLN and Mia-vector cells were treated with fluorescence-labelled anti-EGFR Ab/FcBP-LGA-PEI/miR-198 mimics NPs or LGA-PEI/miR-198 mimics NPs for cell binding and internalization assays. We found that only anti-EGFR Ab/FcBP-LGA-PEI/miR-198 mimics NPs showed a highest fluorescence intensity in Mia-MSLN cells as compared with other groups in both cell binding (Figure 5A) and cell internalization assays (Figure 5B), indicating that anti-EGFR Ab/FcBP-LGA-PEI/miR-198 mimics NPs target to high-EGFR-expressing Mia-MSLN cells. For the control purpose, we added free anti-EGFR Ab to LGA-PEI/miR-198 mimics NPs, which showed no difference of cell binding efficiency between free anti-EGFR Ab to LGA-PEI/miR-198 mimics NPs and LGA-PEI/miR-198 mimics NPs in both Mia-MLSN and Mia-vector cells, while anti-EGFR Ab/FcBP-LGA-PEI/miR-198 mimics NPs still showed a highest cell binding efficiency among all other groups (Figure 5C). We also performed more control experiments by using a non-relevant Ab, anti-HIV gp120 Ab. Either free HIV gp120 Ab added into LGA-PEI/miR-198 mimics NPs or HIV gp120 Ab bound to FcBP-LGA-PEI/miR-198 mimics NPs did not affect the cell binding and internalization efficiency of the NPs in Mia-MSLN and Mia-vector cells, while only anti-EGFR Ab bound to FcBP-LGA-PEI/miR-198 mimics NPs showed a highest cell binding (Figure 6A) and internalization efficiency (Figure 6B) in Mia-MSLN cells (high expression of EGFR), but not in Mia-vector cells (low expression of EGFR). To confirm the functional gene delivery by the FcBP-LGA-PEI polymer, we loaded FcBP-LGA-PEI with GFP-gene-containing plasmid DNA to form NPs and bound anti-EGFR Ab to the NP for active-targeting delivery into Mia-MSLN cells, which have high expression of EGFR. For the control, non-relevant Ab, HIV gp120 Ab, was used to bind to FcBP-LGA-PEI/GFP plasmid DNA NPs. Mia-MSLN cells were treated with anti-EGFR Ab/FcBP-LGA-PEI/GFP plasmid NPs or HIV gp120 Ab/FcBP-LGA-PEI/GFP plasmid NPs for 20 h, and fluorescence cells were observed as an indicator of GFP gene delivery and expression. As expected, Mia-MSLN cells treated with anti-EGFR Ab/FcBP-LGA-PEI/GFP plasmid NPs showed many more fluorescence cells as compared with the cells treated with HIV gp120 Ab/FcBP-LGA-PEI/GFP plasmid NPs, indicating that anti-EGFR Ab specifically targets and enhances delivery of the GFP gene into EGFR high-expressing cells (Figure 6C). Furthermore, in PC cell cultures, MSLN scFv-LGA-PEI/GFP plasmid NPs or anti-EGFR Ab/FcBP-LGA-PEI/GFP plasmid NPs had a higher transfection efficiency in MSLN-overexpressed cell lines than that in vector control cells, indicating targeted delivery of GFP plasmid DNA, while the transfection efficiency of lipofectamine 3000 and LGA-PEI/GFP plasmid NPs was similar in both cell types, indicating no targeted delivery (Supplementary Figure S3).

### 2.3. MSLN scFv-LGA-PEI and Anti-EGFR Ab/FcBP-LGA-PEI NPs Specifically Deliver NAs into Targeting PC Tumor Tissues in Mouse Models

**Figure 7:**
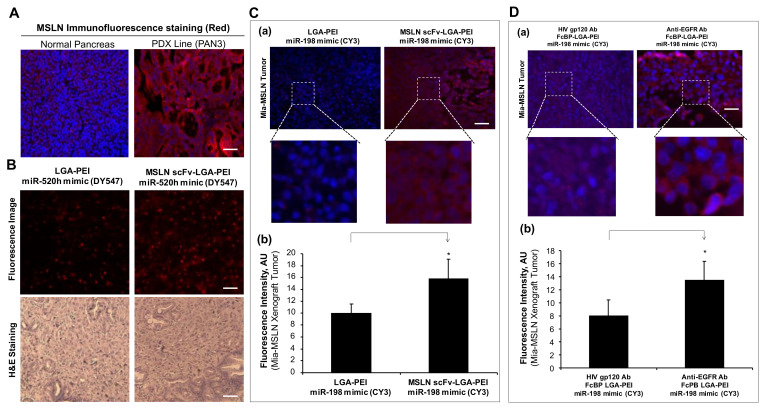
Targeting delivery of MSLN scFv-LGA-PEI or anti-EGFR Ab/FcBP-LGA-PEI nanoparticles (NPs) with nucleic acids to specific cancer cells in mouse models. (**A**) MSLN immunofluorescence staining (red) in normal pancreas tissue and patient-derived xenograft (PDX) pancreatic cancer tissue (PAN3). (**B**) MSLN scFv-LGA-PEI NPs delivering miR-520h mimics into pancreatic cancer tissues in a mouse model. The PDX line (PAN3) was orthotopically implanted into the pancreas of an NSG mouse (NOD scid gamma mouse) for 3 weeks. miR-520h was labeled with DY-547 and loaded into MSLN scFv-LGA-PEI or LGA-PEI polymer to form NPs. MSLN scFv–LGA-PEI (15 μg)/miR-520h mimics (10 μg) NPs or LGA-PEI (15 μg)/miR-520h mimics (10 μg) NPs were randomly injected into the mouse through the tail vein (4 mice/group). The mice were euthanized the next day, and the PAN3 tissues were harvested for immunofluorescence image and H&E staining. (**C**) MSLN scFv-LGA-PEI NPs delivering miR-198 mimics into pancreatic cancer tissues in a mouse model. Mia-MSLN cells (5 × 10^5^ in 50 μL) were orthotopically injected into the pancreas of an NSG mouse for 3 weeks when the xenograft tumor was established. miR-198 mimics were labeled with CY3 and loaded into MSLN scFv-LGA-PEI or LGA-PEI polymer to form NPs. MSLN scFv–LGA-PEI (15 μg)/miR-198 mimics (10 μg) NPs or LGA-PEI (15 μg)/miR-198 mimics (10 μg) NPs were randomly injected into the mouse through the tail vein (4 mice/group). The mice were euthanized the next day, and mouse tumors were harvested for fluorescence analysis. (**a**) Fluorescence macroscopic images. (**b**) Red fluorescence intensity from fluorescence macroscopic images. * *p* < 0.05. (**D**) Anti-EGFR Ab/FcBP-LGA-PEI NPs delivering miR-198 mimics into pancreatic cancer tissues in a mouse model. Mia-MSLN cells (5 × 10^5^, 50 μL) were orthotopically injected into the pancreas of an NSG mouse for 3 weeks. NPs were prepared with 10 μg Cy3-labelled miR-198 mimics 15 μg LGA-PEI and 4 μg FcBP-LGA-PEI polymer with anti-EGFR Ab or HIV gp120 Ab. Anti-EGFR Ab/FcBP-LGA-PEI/miR-198 mimics NPs or HIV gp120 Ab/FcBP-LGA-PEI/miR-198 mimics NPs (200 μL) were randomly injected into the tumor-bearing mice through the tail vein (4 mice/group). The mice were euthanized the next day, and the Mia-MSLN tumor tissues were harvested for fluorescence image (**a**) and fluorescence intensity analysis (**b**). * *p* < 0.05. Scale bar (50 µm).

Recently, we developed many PDX lines from human PC surgical specimens. One of the PDX lines (PAN3) was used in the current study. PDX3 (PAN3) showed high expression of MSLN as compared with the normal human pancreas tissue by immunofluorescence staining (Figure 7A). To determine whether MSLN scFv-LGA-PEI NPs with NAs could target MSLN-expressing cancer tumor tissues in vivo, we orthotopically implanted the PDX line (PAN3) into the pancreas of an NSG mouse (NOD scid gamma mouse) for 3 weeks, when the tumor was established. miR-520h labeled with DY-547 was loaded into the MSLN scFv-LGA-PEI or LGA-PEI polymer to form NPs. MSLN scFv–LGA-PEI/miR-520h mimics NPs or LGA-PEI/miR-520h mimics NPs were randomly injected into the mouse through the tail vein (*n* = 4 for each group). The mice were euthanized 1 day later, and the PAN3 tissues were harvested for immunofluorescence image and H&E staining. The mouse treated with MSLN scFv-LGA-PEI/miR-520h mimics NPs showed stronger red fluorescence in the PAN3 tumor than that treated with the LGA-PEI/miR-520h mimics NPs did, indicating MSLN scFv-LGA-PEI can deliver more RNA into the tumor tissue with high MSLN expression than LGA-PEI does (Figure 7B). In addition, a Mia-MSLN cell-line-based xenograft mouse model was also used to determine the targeting delivery of MSLN scFv-LGA-PEI NPs with NAs. Mia-MSLN cells (5 × 10^5^ in 50 μL) were orthotopically injected into the pancreas of an NSG mouse for 3 weeks, when the tumor was established. miR-198 mimics labeled with CY3 was loaded into MSLN scFv-LGA-PEI or LGA-PEI polymer to form NPs. MSLN scFv-LGA-PEI/miR-198 mimics NPs or LGA-PEI/miR-198 mimics NPs were randomly injected into the mouse through the tail vein (*n* = 4 for each group). The mice were euthanized in next day, and mouse Mia-MSLN cell-derived tumors were harvested for fluorescence analysis. The mouse treated with MSLN scFv-LGA-PEI/miR-198 mimics NPs showed an enhanced red fluorescence in the Mia-MSLN tumors compared with the mouse treated with LGA-PEI/miR-198 mimics NPs, suggesting that MSLN scFv-LGA-PEI polymer more efficiently delivers RNA into the pancreatic tumors with high expression of MSLN (Figure 7C).

Furthermore, we also determined the targeting delivery of anti-EGFR Ab/FcBP-LGA-PEI miR-198 mimics NPs into Mia-MSLN cell-derived xenografts in the mouse model. Mia-MSLN cells were orthotopically injected into the pancreas of a NSG mouse for 3 weeks, when the tumor was established. Anti-EGFR Ab/FcBP-LGA-PEI miR-198 mimics NPs or HIV gp120 Ab/FcBP-LGA-PEI/miR-198 mimics NPs as the control randomly injected the tumor-bearing mouse through tail vein (*n* = 4 for each group). The mice were euthanized the next day, and the Mia-MSLN tumor tissues were harvested for fluorescence image analysis. The mouse treated with anti-EGFR Ab/FcBP-LGA-PEI/miR-198 mimics NPs showed significantly higher fluorescence intensity in the Mia-MSLN tumors as compared with that in mice treated with HIV gp120 Ab/FcBP-LGA-PEI/miR-198 mimics NPs, indicating that anti-EFGR Ab guides NP delivery of nucleic acids into Mia-MSLN tumors with high expression of EGFR (Figure 7D).

## 3. Discussion

In the current study, we extended our previously developed new LGA-PEI polymer as an improved NP platform for delivering TNAs to active-targeting delivery systems by covalent conjugation of MSLN scFv Ab fragment or FcBP for binding any full Ab (IgG). These Ab-guided LGA-PEI delivery systems are capable of effectively loading TNAs including plasmid DNA, mRNA, and miRNAs to form stable and functionalized NAs. MSLN scFv-LGA-PEI NPs with TNAs significantly improve their binding and internalization as well as gene expression in PC cell lines with high expression of MSLN, a cell surface marker, in both cell culture and mouse models. As a model, we used an FDA-approved humanized anti-EGFR monoclonal Ab (Cetuximab, Eli Lilly and Company, Indiana, USA), binding to our new FcBP-LGA-PEI polymer, which effectively loads TNAs, forms functionalized NPs, and specifically targets PC cell lines with high expression of ERGR in vitro and in vivo. These results demonstrate that two Ab-guided NP delivery platform technologies built up from our novel LGA-PEI polymer may have great potential for clinical applications of the active-targeting therapy with TNAs for PC and other types of cancers with unique and specific cell surface markers.

The targeting drug delivery system could significantly improve the efficacy of the medication, while also reducing side effects as compared with the traditional drug delivery systems such as oral ingestion and intravenous administration [29]. In fact, the NP-based drug delivery system is the passive-targeting drug delivery for cancers and some other diseases through the mechanism of the EPR effect [12,13]. Furthermore, an NP-based drug delivery system can become the active-targeting drug delivery through conjugation of cell-specific ligands or Abs, which allow the NP to bind specifically to the cell that has the complementary receptor or unique cell surface biomarkers. NP-based active targeting can enhance the effects of passive targeting to make the NP more specific to a target site [29]. MSLN, a tumor-associated antigen, is a clinically validated target for immunotherapy and other treatment strategies for PC, mesothelioma, and ovarian cancer, including immunotoxins, vaccines, chimeric monoclonal Abs, Ab-drug conjugates, and chimeric antigen receptor (CAR) T-cell therapy [72]. For the first time, we developed NP-based active-targeting delivery of TNAs specifically towards MSLN-expressing PC cells. We produced a MSLN Ab scFv (SS1) based on the published information [70,71]. In fact, scFv (SS1) was developed by optimization of amino acid sequences from a parental anti-MSLN scFv isolated from a phage display library and showed at least five times the binding affinity for MSLN than the parental Ab scFv [70,71]. MSLN scFv (SS1) was more effectively internalized into cells after binding to the cell surface MSLN as compared with anti-MSLN monoclonal Ab K1 [70,71,73]. MSLN scFv (SS1) was used for the development of a recombinant immunotoxin [74,75] and CAR-T therapy [76] that targets MSLN-expressing cancer cells in preclinical models and clinical trials. In the current study, we covalently linked MSLN scFv to the LGA-PEI polymer though the Mal-PEG-NHS polymer as a hetero-bifunctional crosslinker. Primary amine of PEI attacks ester bond between PEG and NHS groups of the Mal-PEG-NHS polymer, thus replacing the NHS group to conjugate Mal-PEG to the primary amine of PEI of the LGA-PEI polymer. The hydrosulfyl group (-SH) (thiol) of cysteine in MSLN scFv is also ready to react the C=C double bond of the maleimide group of the Mal-PEG-PEI-LGA under nitrogen to form a stable S-C bond (click reaction) for linking MSLN scFv to Mal-PEG-PEI-LGA.

It is well known that hydrophilic polymer PEG is often used to modify PEI for reducing the surface charge, while increasing solubility of the PEI-based NPs in vivo. Such a modification strategy is termed “stealth technology”; thereby, PEGylation of PEIs could diminish the interaction of the NPs with blood components, reduce the uptake by macrophages, and increase blood circulation time for the effective delivery [26,77]. The PEG-PEI copolymer showed good transfection and low cytotoxicity compared with PEI alone [78]. An optimal amount of PEG for PEI modification is important because heavy PEGylation of PEI could reduce the TNA-binding capacity of the polymer [78]. Further functionalization of the PEI polymer is to combine the PEG stealth technology with the use of ligands and Abs (scFv or full Abs) for the active-targeting delivery though a hetero-bifunctional crosslinker such as α-maleimide-ω-N-hydroxysuccinimide ester polyethylene glycol (Mal-PEG-NHS ester) [79,80]. Thiol–ene click reaction including thiol–ene radical and thiol Michael addition reaction is one of the most commonly used methods for preparing peptide–polymer conjugates [81]. In the current study, the maleimide of MAL-PEG-NHS can react with a free thiol group of cysteine residue of MSLN scFv by the Michael addition reaction, while the NHS can react with the primary amine of the PEI-LGA polymer. It is believed that the Michael addition reaction is of high efficiency and selectivity, and it could be achieved under mild conditions without interference from other functionalities of the polymer [81]. ScFv consists only of the variable regions of immunoglobulins and has the same targeting specificity of the parental form of full Ab. Because of the small size, scFv has some advantages for the active-targeting delivery system such as more conjugation, low immunogenicity, and more efficient tissue penetration compared with full Abs [82,83]. Modified branched PEI for the delivery system should remain its positively charged amine groups (primary, secondary, and tertiary), which enable effective electrostatic binding and condensation of negatively charged TNAs. Secondary and tertiary amines of modified PEI are also critical for its buffering capacity and polymer swelling at the acidic pH of the endosomes [84]. Our MSLN scFv-LGA-PEI copolymer effectively loaded double-stranded plasmid DNA, single-stranded RNA and miRNA mimics, and formed NPs with sizes of around 100 nm, which are ready for active-targeting delivery.

In the current study, we also developed a new nanoplatform technology based on our LGA-PEI polymer, which covalently linked to a small cyclic peptide FcBP (CGGGGDCAWHLGELVWCTGGGGC) through the hetero-bifunctional crosslinker, Mal-PEG-NHS ester. Full Abs can be tagged non-covalently to FcBP-LGA-PEI NPs with TNAs, resulting in higher affinity for specifically targeting cells and tumor tissues in vivo. It is a multifunctional and versatile nanoplatform, which can be loaded with different types of full Abs for molecular diagnosis or treatment, separately or simultaneously. Similar to gram-positive bacteria protein A/G, the 13-mer core FcBP (Fc-III, DCAWHLGELVWCT) was discovered by the bacteriophage display technique and showed a high affinity, binding to a common site between the CH2 and CH3 domains of human IgG Fc [52]. Previous studies showed that FcBP (Fc-III) is ready to covalently conjugate to the PEG linker for variable applications, such as improved technology for isolation and purification of Abs and Ab-guided NP delivery systems [85,86]. Abs captured by FcBP fully retain their structure and function. Experimental data showed that FcBP (Fc-III) has a high affinity for human, donkey, and rabbit Abs (IgG1 and IgG2), and a weak affinity for mouse, rat, and goat Abs [85,87], except one study showed that FcBP (Fc-III) also binds to mouse IgG [88]. In our study, we added four extra glycine residues at each end of the 13-mer core FcBP (Fc-III) for more space and the conformational flexibility to bind to the Fc of full Abs. This strategy was successfully utilized by previously studies, in which modified FcBP was genetically incorporated into the protein-based NPs [89,90]. In addition, we added one cysteine at both ends of the FcBP for its reaction and oriented linkage with the Mal-PEI linker without affecting functional cyclic structure of the FcBP though internal S-S bond of two cysteine residues [52]. For the functional test of our FcBP-LGA-PEI copolymer, we loaded a human anti-EGFR Ab (Cetuximab) as a model system because we demonstrated that MSLN increased EGFR expression in PC cell lines (Figure 3A) and Cetuximab can significantly inhibit cell proliferation in a subset of PC cell lines with high expression of both MSLN and EGFR [53]. Although EGFR gene amplifications and mutations in PC are rare [91,92], the overexpression of EGFR is observed in 9–90% of PC tissues [93,94,95] and it has been associated with higher-stage and more aggressive tumors, worse clinical outcomes, poor survival, and metastasis of PC [53,96,97]. Thus, EGFR becomes a critical target for the treatment of PC [54,95]. Cetuximab (Erbitux) is an anti-EGFR chimeric (mouse/human) monoclonal Ab, which was initially approved for the treatment of certain types of cancers by the FDA in 2004 [98]. Cetuximab has been conjugated to variable NPs such as PLGA [99] and gold particles [100,101] for imaging and/or active-targeting delivery for different types of cancers. It was also covalently conjugated to low-molecular-weight branched PEI (~4−10 kDa) through a bifunctional linker PEG, and Cetuximab-functionalized PEG-PEI NP with DNA or siRNA showed its capability of binding and uptaking into the EGFR-overexpressing cells [102]. For the NP formation, we first loaded TNAs such as plasmid DNA to the FcBP-LGA-PEI copolymer, and then anti-EGFR Ab (Cetuximab) was added to the NP preparation to form the final anti-EGFR Ab/FcBP-LGA-PEI NPs, whose size was confirmed to be around 100 nm (Figure 2C). Usually, the size of a full IgG Ab is about 4.9 nm with the molecular weight of 150 kDa, which is much bigger than scFv (2.6 nm with 25 kDa) [35]. The optimal incorporation condition of full Ab or scFv into the LGA-PEI-based NPs should be considered differently.

The PEI-based NP delivery system has a different cellular uptake mechanism compared with the liposome-based delivery system. Liposome NPs enter cells by clathrin-mediated endocytosis, whereas PEI NPs are taken up by both mechanisms of clathrin-mediated endocytosis and caveolae-mediated endocytosis, which are associated with the lysosomal escape mechanism [70,103]. Thus, PEI is more efficient for TNA delivery than liposome. Ab/cell surface receptor-medicated endocytosis could be involved in multiple mechanisms including caveolin-dependent endocytosis and clathrin-independent mechanisms such as phagocytosis and micropinocytosis. It is believed that clathrin-dependent endocytosis is the predominant mechanism for the internalization of cell-surface receptors [103,104]. Ligand-based targeting delivery is much more efficient than non-ligand-based polymer delivery systems [105,106,107]. Indeed, our two active-targeting NP delivery systems with MSLN scFv or anti-EGFR Ab (Cetuximab) showed a significant enhancement of NP binding and internalization as well as gene expression in MSLN- or VFGF-expressing PC cell lines as compared with NPs without MSLN scFv or anti-EGFR Ab (Figure 3, Figure 4, Figure 5 and Figure 6). Our LGA-PEI-based delivery systems showed consistent results when they were loaded with either miRNA mimics or GFP plasmid DNA as a model of TNAs. When a human PC cell line (Mia-MSLN) with high expression of MSLN was orthotopically injected into the pancreas of the immune-deficient mouse, the cells proliferated and formed the tumor tissues. We observed intravenous administration of MSLN scFv-LGA-PEI NPs or anti-EGFR Ab/FcBP-LGA-PEI NPs loaded with fluorescence-labelled TNAs had a significantly higher delivery efficiency into Mia-MSLN tumors than non-targeting NPs did in this cell-line-based xenograft mode in the mouse (Figure 7C,D).

Although the cell-line-based xenograft model is useful for the study of the molecular pathway and “the proof of concept” therapies, when used for drug discoveries, it may not reliably predict clinical efficacy [108]. This has led to the development of models by directly engrafting patient-derived tumor tissues into immunodeficient mice in order to retain the histopathologic features and molecular characteristics of the original tumor [109]. The PDX model of PC retains a greater proportion of stromal components and genetic characteristics of the human tumor specimens from which they were derived, develops regional and distant metastases, and is more predictive of patient response than the traditional cell-line-based xenograft model [110,111]. Thus, the PDX model is very useful for therapeutic efficacy, drug and biomarker discovery, and to study tumor biology and personalized medicine. For example, several recent pre-clinical studies were successfully performed to evaluate drug efficacy in subcutaneous PDX models of PC [112,113]. For classical chemotherapeutic drugs, PC PDX models based on clinical specimens can predict 90% drug sensitivity and 97% drug resistance [114]. In the past several years, we established many PDX lines from surgical specimens of patients with PC. In the current study, we selected the PDX line (PAN3) with high expression of MSLN. We observed that NSLN scFv-LGA-PEI NPs with fluorescence-labelled miRNA mimics have a higher delivery efficiency than non-targeting NPs do in the PDX model in mice, which is more clinically relevant to the cell-line-based xenograft model.

Although polymer-based NP delivery systems for TNAs have great potential for broad clinical applications of treatment and diagnosis of many types of cancers and other diseases, many challenges and hurdles of these new technologies still exist. For examples, optimal design of the size, shape, and surface charge of NPs is difficult to define because it may be different for each individual type or subtype of cancers. In addition, tumor microenvironments such as cell density, cell type, molecular profiling, pH condition, hydrostatic pressure, and diffusion rate of NPs are critical factors, which significantly influence the delivery efficiency and therapeutic efficacy of NP medicine. Furthermore, it is difficult to study pharmacokinetics of the multi-component NP medicine in humans [115,116]. In the current study, our LGA-PEI/NA NPs were labelled by a fluorescence dye, and used in both in vitro and in vivo experiments. For animal studies, fluorescence labelled NPs were not sensitive for the whole-body imaging. For a future study, we could load luciferase gene DNA into the LGA-PEI or Ab-linked LGA-PEI NPs, which can be used for animal experiments because chemiluminescence-based technology is more sensitive for the whole-body imaging. This method could demonstrate bio-distribution and targeted delivery of our LGA-PEI-based NP delivery systems. In the animal studies, we chose one day after NP i.v. injection for animal euthanization because this time frame was sensitive to NPs entering into cells based on our in vitro experiments. In a future study, a time cause of delivery efficiency such as 6, 12, 24, and 48 h after NP injection could generate meaningful data of delivery kinetics. Furthermore, dose dependent studies will be further studied as well.

In summary, we developed two active-targeting NP delivery technologies for TNAs from our existing novel LGA-PEI polymer. Our first technology was the covalent linkage of MSLN scFv to LGA-PEI through a bifunctional linker Mal-PEG-NHS; the MSLN scFv-LGA-PEI copolymer can efficiently load TNAs including miRNA mimics and plasmid DNA as well as mRNA to form NPs with sizes around 100 nm. Our second technology was the covalent linkage of FcBP (modified Fc-III) to LGA-PEI through Mal-PEG-NHS; the FcBP-LGA-PEI copolymer can also effectively load TNAs to form NPs and bind to Fc region of any human IgG (Abs). As a model system, we successfully linked anti-EGFR Ab (Cetuximab) to the FcBP-LGA-PEI copolymer with TNAs. We have produced high quality NPs from our polymers (LGA-PEI, MSLN scFv-LGA-PEI, anti-EGFR Ab/FcBP-LGA-PEI) with different forms of TNAs based on the analysis of the polydispersity index (PDI) of NPs [117,118,119,120]. Both MSLN scFv-LGA-PEI and anti-EGFR Ab/FcBP-LGA-PEI systems are more effective and preferentially deliver TNAs into PC cells with the high expression of MSLN or EGFR, respectively, in vitro and in vivo. More importantly, we showed our new technology works in the PDX model, which is more clinically relevant than other models. Our active-targeting NP-delivery systems are a platform technology, which may have great potential for clinical applications as a new generation of delivery systems for TNAs for the treatment or molecular diagnosis of different types of cancers or other diseases.

In the future, the therapeutic efficacy of these two technologies for pancreatic cancer will be performed in clinically relevant animal models such as PDXs. We expect that these active-targeting deliveries of TNAs are more effective at inhibiting tumor growth and metastasis as well as at improving the overall survival of tumor-bearing animals as compared with passive-targeting delivery or other non-targeting delivery systems. Since our active-targeting delivery systems are the platform technology, they can be extended to other cancer types or diseases for the development of new treatment and molecular diagnosis strategies. These technologies could also be used for vaccine development. The ultimate goal of the project is to apply our technologies to broad clinical applications through extensive preclinical studies and clinical trials including efficacy, pharmacokinetics, and short-term and long-term toxicity.

## 4. Material and Methods

### 4.1. Materials

PLGA (50:50) (12 kDa–16 kDa, lactide:glycolide 50:50 mol/mol, i.v. 0.50–0.65) was obtained from Polysciences Inc. (Warrington, PA, USA). Branched PEIs with average MW ~25 kDa were obtained from Sigma-Aldrich (St. Louis, MO, USA). N-succinimidyl-sacetylthiopropionate (SATP) was obtained from ThermoFisher Scientific (Grand Island, NY, USA). Maleimide-PEG3500-NHS was obtained from Jenkem Technology (Shanghai, China). Green fluorescence protein (GFP)- or red fluorescence protein (RFP)-containing DNA plasmids (5 kb and 8 kb, respectively) were prepared by Aldevron (Fargo, ND, USA). Custom miRIDIAN mimic (GE Healthcare Dharmacon Inc., Colorado, USA) for human has-miR-198 (double stranded RNA) was synthesized with Cy3 fluorescent tag on the passenger strand. The mature sequence of miR-198 is 22 bp: 5′ GGUCCAGAGGGGAGAUAGGUUC. Custom miRIDIAN mimics (Thermo Fisher Scientific BioSciences, Dublin, Ireland) for miR-520h (double stranded RNA) was synthesized with DY-547 fluorescent tag on the passenger strand. The mature sequence of miR-520h is 22 bp: ACAAAGUGCUUCCCUUUAGAGU. FcBP was synthesized by Biomatik (Wilmington, DE, USA) with a sequence (N to C): CGGGGDCAWHLGELVWCTGGGGC (23aa). MSLN scFv (SS1) gene sequence was designed and synthesized based on the published information [70,71]; it was cloned into a plasmid vector and expressed in yeast (*Pichia pastoris* X33 strain). Synthesized 255-bases modified mRNA (single-stranded RNA) from a part of X-inactive specific transcript fragment (XIST containing 255 nucleotides) gene (mmRNA-XIST255) and control mmRNA-255 were synthesized in the RNA Core, the Houston Methodist Research Institute (Houston, TX, USA).

### 4.2. Preparation of MSLN scFv-LGA-PEI and FcBP-LGA-PEI

Synthesis of the LGA-PEI polymer was described in our previous publication [28]. The Ab fragment MSLN scFv or FcBP was conjugated to LGA-PEI through bi-functional PEG (Maleimide-PEG3500-NHS ester) (Figure 1A and Figure S1). First, Mal-PEG-NHS was combined with LGA-PEI at a desired molar ratio to form maleimide-functionalized LGA-PEI (LGA-PEI-PEG-Mal). Second, MSLN scFv or FcBP was modified with SATP to convert primary amines into thiols by following the instructions provided by Thermo Fisher Scientific. Briefly, 0.4 mg MSLN scFv or FcBP was combined with 8 mg of SATP in PBS/DMSO at 25 °C for 30 min. Then, the desired product was purified using a PD-10 desalting column. The SATP groups were deacetylated using hydroxylamine and EDTA and purified with a PD-10 column. To produce MSLN scFv-LGA-PEI or FcBP-LGA-PEI, 0.2 mg of thiol-modified MSLN scFv or FcBP was combined with 50 mg of maleimide-functionalized LGA-PEI (or at a desired molar ratio) at 25 °C for 20 h. The sulfhydryl group (-SH) of thiol-modified MSLN scFv or FcBP is added to the C=C double bond of the maleimide group in Mal-PEG-PEI-LGA to form a stable S-C bond. The amount of MSLN scFv or FcBP conjugated to LGA-PEI was quantified with the Micro BCA method (Thermo Fisher Scientific, Waltham, MA, USA) and spectrophotometry.

### 4.3. MSLN scFv-LGA-PEI or FcBP-LGA-PEI Polymer and NA Loading

NPs of MSLN scFv-LGA-PEI or FcBP-LGA-PEI polymer and NAs, including plasmid DNA, mmRNA, and miRNA mimics, were prepared at different polymer to NA ratios by adding the NA solution to the water solution of polymer, and vortexing for 5 s. For example, 2 μg of plasmid DNA in 25 μL of water was added to 5 μg of the MSLN scFv-LGA-PEI or FcBP-LGA-PEI polymer in 25 μL water. These NP suspensions were kept at room temperature for 30 min before use without any further treatment. For the anti-EGFR Ab/FcBP-LGA-PEI system, we loaded the FcBP-LGA-PEI polymer with NAs to form the FcBP-LGA-PEI/NA complex; then, we added anti-EFGR Ab in the FcBP-LGA-PEI/NA complex to form the final anti-EFGR Ab/FcBP-LGA-PEI/NA NPs.

### 4.4. Particle Size and Zeta Potential Measurements

The size distributions of polymer/NA NPs were determined with dynamic light scattering using Zetasizer Nano ZS90 (Malvern Panalytical Ltd., Worcestershire, UK). The zeta potential of the NPs was measured with Zetasizer Nano ZS90 at a temperature of 25 °C. Some of the NPs were also analyzed with scanning electronic microscopy (SEM) and energy-dispersive spectroscopy (EDS).

### 4.5. Cell Binding and Internalization Assays

Different types of NPs formed with our polymers and NAs were labelled with fluorescein Cy3 or DY-547 in two types of genetically engineered PC lines, Mia-PaCa 2 with stable transfection of human MSLN gene (Mia-MSLN cells) and Mia-PaCa 2 with stable transfection of empty vector cells (Mia-vector), which were previously established in our lab [32]. For cell-binding assay, Mia-vector and Mia-MSLN cells were seeded onto 8-well Chamber slides (Lab-Tek II, Thermo Fisher Scientific, Waltham, MA, USA), incubating overnight to ensure cell attachment. Fluorescein-labeled NPs were added the chamber and incubated for 2 h at 4 °C, followed by washing twice with PBS and fixation for 15 min with 4% p-formaldehyde. The cell binding efficiency of the NPs to the cells was analyzed with fluorescence images taken with an Olympus IX51 microscope and fluorescence intensity analysis with ImageJ software (NIH). For the cell internalization assay, fluorescein-labeled NPs were added to the chamber with Mia-NSLN or Mia-vector cells and incubated for 2 h at 4 °C, and then the cells were replaced with fresh culture medium and cultured at 37 °C overnight. The cell internalization efficiency of the NPs to the cells was analyzed with fluorescence images and fluorescence intensity analysis. For experiments (Figure 5 and Figure 6A,B), miR-198 mimics (1 µg) labeled with a fluorescence dye Cy3 were loaded into either LGA-PEI (1.5 µg) or anti-EFGR Ab/FcBP-LGA-PEI (1.5 µg) NPs. Anti-EGFR Ab/FcBP-LGA-PEI/miR-198 mimics NPs or LGA-PEI/miR-198 mimics control NPs were added into the cell culture of Mia-MSLN cells and Mia-vector control cells at 70% confluence for the binding assay or internalization assay.

### 4.6. Delivery of GFP Gene In Vitro

Different types of NPs formed with MSLN scFv-LGA-PEI, FcBP-LGA-PEI, Ab, and GFP-containing plasmid DNA were added into the cell culture of Mia-MSLN or Mia-vector cells for 24 or 48 h. The transfection rate of GFP expression was evaluated by fluorescence image analysis. For experiments (Figure 4C), LGA-PEI/GFP plasmid DNA NPs NPs were prepared by mixing 1 μg DNA in 12.5 mL H_2_O with 3.5 μg LGA-PEI polymer in 12.5 μL H_2_O; MSLN scFv-LGA-PEI/GFP plasmid DNA NPs were prepared by mixing 1 μg DNA in 12.5 mL H_2_O with 0.86 ug MSLN scFv-LGA-PEI plus 3.5 μg LGA-PEI polymers in 12.5 μL H_2_O (LGA-PEI). NPs were kept at room temperature for 30 min before use. For experiments (Figure 6C), anti-EGFR Ab/FcBP-LGA-PEI/GFP plasmid DNA NPs were prepared by mixing 1 μg DNA in 12.5 µL H_2_O with 0.86 µg FcBP-LGA-PEI plus 3.5 μg LGA-PEI polymers in 12.5 μL H_2_O. For controls, HIV gb120 Ab/FcBP-LGA-PEI/GFP plasmid DNA NPs were prepared by mixing 1 μg DNA in 12.5 µL H_2_O with 0.86 µg FcBP-LGA-PEI plus 3.5 μg LGA-PEI polymers in 12.5 μL H_2_O. NPs were kept at room temperature for 30 min. HIV gp120 Ab (0.2 µg) or anti-EGFR Ab (0.2 µg) was added to the respective NP solution and kept at room temperature for 10 min more before use. The NPs in 25 mL H_2_O diluted with 200 mL plain DMEM medium (without FBS) were then added to 10k-15k Mia-MSLN cells/well growing in 24-well plates. After 3 h, the NPs/DMEM medium was replaced with 500 mL fresh DMEM culture medium (with 15% FBS) and the cells were incubated for another 24 h before taking fluorescence images. Gene expression was measured by observing GFP-positive cells under a fluorescent microscope.

### 4.7. Delivery of Nucleic Acids in Mouse Models

The following animal work was approved by the Office for Protection from Research Risks and Animal Welfare Act guidelines under an animal protocol approved by the Baylor College of Medicine Institutional Animal Care and Use Committee. For the patient-derived xenograft (PDX) model, the PDX line (PAN3) was orthotopically implanted into the pancreas of an NSG mouse (NOD scid gamma mouse) for 3 weeks, when the tumor was established. Fluorescence-labeled NPs were injected to the mouse through the tail vein (4 mice/group). The mice were euthanized 1 day later, and the PAN3 tissues were harvested for immunofluorescence image and H&E staining. For the cell-line-based xenograft model, Mia-MSLN cells (5 × 10^5^ in 50 μL) were orthotopically injected into the pancreas of an NSG mouse for 3 weeks, when the tumor was established. Fluorescence-labeled NPs were injected into the mouse through the tail vein (4 mice/group). The mice were euthanized the next day, and mouse tumors were harvested for fluorescence analysis. Both male and female mice (with age 8–12 weeks old and body weight 20–30 g) were used in the experiments.

### 4.8. Statistical Analysis

All data are presented as the mean ± SEM. Nanoparticle delivery experiments in vitro were repeated five times for each group. Mouse experiments with four mice for each group were performed. Differences among three or more groups were analyzed with one-way analysis of variance (ANOVA). Student t-test was used to compare the two groups. A *p* value < 0.05 was regarded as significant.

## Data Availability

Data is contained within the article and Supplementary Material.

## Supplementary Materials

The following are available online at https://www.mdpi.com/article/10.3390/ph14090841/s1, Table S1. Therapeutic nucleic acids approved for clinical applications; Table S2. Mesothelin expression in different types of cancers; Figure S1. Synthesis of FcBP-LGA-PEI polymer and nanoparticle (NP) formation with antibody (Ab) and nucleic acids; Figure S2. Gel retardation assay to determine the nucleic acid load efficiency of our polymers (LGA-PEI, MSLN scFv-LGA-PEI and anti-EGFR Ab/FcBP-LGA-PEI); Table S3. Nanoparticle sizes for different ratios of MSLN scFv-LGA-PEI and LGA-PEI (total 5 µg) and the same amount of the plasmid DNA (2 µg); Table S4. Nanoparticle sizes for different ratios of MSLN scFv-LGA-PEI and LGA-PEI (total 1.5 µg) and the same amount of miR-198 mimics (1 µg); Table S5, Nanoparticle sizes for different ratios of anti-EGFR Ab/FcBP-LGA-PEI and LGA-PEI (total 5 µg) and the same amount of plasmid DNA (2 µg); and Figure S3. Transfection efficiency of green fluorescence protein (GFP) gene mediated by different polymers (LGA-PEI, MSLN scFv-LGA-PEI and anti-EGFR Ab/FcBP-LGA-PEI) and commercial lipofectamine 3000 in MSLN overexpression and vector control cells in vitro.
