# Supplementary material for: Two Antibody-Guided Lactic-co-Glycolic Acid-Polyethylenimine (LGA-PEI) Nanoparticle Delivery Systems for Therapeutic Nucleic Acids
Source: Pharmaceuticals (Basel). 2021 Aug 25;14(9):841. doi: 10.3390/ph14090841 (PMC8470087; doi:10.3390/ph14090841)
Supplement: Supplementary file 1 [file pharmaceuticals-14-00841-s001.zip › pharmaceuticals-1285832-supplementary.pdf]

Article

Two Antibody-Guided Lactic-co-Glycolic Acid-Polyethylenimine (LGA-PEI) Nanoparticle Delivery Systems for Therapeutic Nucleic Acids

SUPPLEMENTARY MATERIALS

Table S1. Therapeutic nucleic acids approved for clinical applications

Name	Mechanism	Treatment	Approval
Fomivirsen (trade name Vitravene)	Antisense oligonucleotide	Cytomegalovirus retinitis (CMV)	FDA approval in 1998
Gendicine	Recombinant adenovirus-wildtype-p53 (rAd-p53)	Tumors which have mutated p53 genes	China approval in 2003
Pegaptanib (trade name Macugen)	Pegylated anti-vascular endothelial growth factor (VEGF) aptamer	Neovascular (wet) age-related macular degeneration (AMD)	FDA approval in 2004
Cambiogenplasmid (trade name Neovasculgen)	Plasmid-CMV-VEGF	Peripheral artery disease	Russia approval in 2011
Alipogene tiparvovec (trade name Glybera)	Adeno-associated virus serotype 1 (AAV1) viral vector- human lipoprotein lipase (LPL) gene	Lipoprotein lipase deficiency (LPLD), severe pancreatitis	Europe approval in 2012
Mipomersen (trade name Kynamro)	Antisense oligonucleotide for apolipoprotein B-100	Homozygous familial hypercholesterolemia	FDA approval in 2013
Defibrotide (trade name Defitelio)	Mixture of single-stranded oligonucleotides<br>Mechanism of action is poorly understood, protecting the endothelium lining blood vessels from damage by fludarabine.	Veno-occlusive disease of the liver of of people having had a bone marrow transplant.	Europe approval in 2013<br>FDA approval in 2015
Talimogene laherparepvec (trade name Imlygic)	Herpes virus (an oncolytic herpes virus) vector- human GM-CSF gene	Melanoma	FDA approval in 2015
Betibeglogene autotemcel (trade name Zynteglo)	Lentiviral vector- HBB gene<br>HSC ex vivo	Beta thalassemia, a rare blood disorder	FDA approval in 2015
Nusinersen (trade name Spinraza)	Antisense oligonucleotide for	spinal muscular atrophy (SMA)	FDA approval in 2016
Eteplirsen (trade name Exondys 51)	Morpholino antisense oligomer which triggers excision of exon 51 of dystrophy pre-mRNA during pre-mRNA splicing of the dystrophin RNA transcript.	Duchenne muscular dystrophy (DMD)	FDA approval in 2016
Strimvelis	Retrovirus-human adenosine deaminase gene	Adenosine deaminase deficiency (ADA-SCID) (ex vivo gene delivery for CD34+ HSCs)	Europe approval in 2016
Voretigene neparvovec (trade name Luxturna)	AAV2 vector-human RPE65 cDNA with a modified Kozak sequence	Leber's congenital amaurosis, or biallelic	FDA approval in 2017

		RPE65-mediated inherited retinal disease	
Tisagenlecleucel (trade name Kymriah)	A chimeric T cell receptor ("CAR-T") CD19	B-cell acute lymphoblastic leukemia (ALL)	FDA approval in 2017
Axicabtagene ciloleucel (trade name Yescarta)	A chimeric T cell receptor ("CAR-T") CD19	Diffuse large B-cell lymphoma	FDA approval in 2017
Patisiran (trade name Onpattro)	Small interfering RNA for transthyretin	Hereditary transthyretin amyloidosis	FDA approval in 2018
Inotersen (trade name Tegsedi)	Antisense oligonucleotide (ASO) for transthyretin	Hereditary transthyretin amyloidosis	FDA approval in 2018
Onasemnogene abeparvovec (trade name Zolgensma)	AAV9 virus vevctor-SMN1 gene with synthetic promoters	Spinal muscular atrophy (SMA)	FDA approval in 2019
Givosiran (trade name Givlaari)	small interfering RNA (siRNA) for 5-aminolevulinic acid synthase	Acute hepatic porphyria (AHP)	FDA approval in 2019
Leqvio (trade name Inclisiran)	small interfering RNA (siRNA) for Proprotein convertase subtilisin/kexin type 9 (PCSK9)	Hypercholesterolemia	Europe approval in 2020
COVID-19 Vaccine (Pfizer)	miRNA for SARS-CoV-2 spike protein	COVID-19 prevention	FDA approval in 2020
COVID-19 Vaccine (Moderna)	miRNA for SARS-CoV-2 spike protein	COVID-19 prevention	FDA approval in 2020

Table S2. Mesothelin expression in different types of cancers*

Cancer Types	Expression
Epithelial mesotheliomas	100%
**Pancreatic cancer**	**80% - 90%**
Ovarian cancers	80% - 90%
Triple-negative breast cancer	50% - 70%
Lung adenocarcinomas	>50%
Colorectal cancer	>50%
Stomach cancer	>50%
Liver cancer	30% - 40%
Uterine serous carcinoma	>30%
cholangiocarcinoma	>30
Acute myeloid leukemia (AML)	>20%

*Information is obtained from references below:

1. Li M, Bharadwaj U, Zhang R, Zhang S, Mu H, Fisher WE, Brunicardi FC, Chen C, Yao Q. Mesothelin is a malignant factor and therapeutic vaccine target for pancreatic cancer. Mol Cancer Ther. 2008 Feb;7(2):286-96.
2. Kelly RJ, Sharon E, Pastan I, Hassan R. Mesothelin-targeted agents in clinical trials and in preclinical development. Mol Cancer Ther. 2012 Mar;11(3):517-25.
3. Scales SJ, Gupta N, Pacheco G, Firestein R, French DM, Koeppen H, Rangell L, Barry-Hamilton V, Luis E, Chuh J, Zhang Y, Ingle GS, Fourie-O'Donohue A, Kozak KR, Ross S, Dennis MS, Spencer SD. An antimesothelin-monomethyl auristatin e conjugate with potent antitumor activity in ovarian, pancreatic, and mesothelioma models. Mol Cancer Ther. 2014 Nov;13(11):2630-40.
4. Kobayashi K, Sasaki T, Takenaka F, Yakushiji H, Fujii Y, Kishi Y, Kita S, Shen L, Kumon H, Matsuura E. A novel PET imaging using ^{64}Cu-labeled monoclonal antibody against mesothelin commonly expressed on cancer cells. J Immunol Res. 2015;2015:268172.
5. Lv J, Li P. Mesothelin as a biomarker for targeted therapy. Biomark Res. 2019 Aug 23;7:18.
6. Ordonez NG. Value of mesothelin immunostaining in the diagnosis of mesothelioma. Mod Pathol. 2003;16(3):192–7.
6. Ordonez NG. Application of mesothelin immunostaining in tumor diagnosis. Am J Surg Pathol. 2003;27(11):1418–28.
7. Argani P, Iacobuzio-Donahue C, Ryu B, Rosty C, Goggins M, Wilentz RE, Murugesan SR, Leach SD, Jaffee E, Yeo CJ, Cameron JL, Kern SE, Hruban RH. Mesothelin is overexpressed in the vast majority of ductal adenocarcinomas of the pancreas: identification of a new pancreatic cancer marker by serial analysis of gene expression (SAGE). Clin Cancer Res. 2001;7(12):3862–8.
8. Hassan R, Laszik ZG, Lerner M, Raffeld M, Postier R, Brackett D. Mesothelin is overexpressed in pancreaticobiliary adenocarcinomas but not in normal pancreas and chronic pancreatitis. Am J Clin Pathol. 2005;124(6):838–45.
9. O'Hara MH, Stashwick C, Plesa G, Tanyi JL. Overcoming barriers of car T-cell therapy in patients with mesothelin-expressing cancers. Immunotherapy. 2017;9(9):767–80.
10. Chang K, Pastan I, Willingham MC. Frequent expression of the tumor antigen CAK1 in squamous-cell carcinomas. Int J Cancer. 1992;51(4):548–54.
11. Tchou J, Wang LC, Selven B, Zhang H, Conejo-Garcia J, Borghaei H, Kalos M, Vondeheide RH, Albelda SM, June CH, Zhang PJ. Mesothelin, a novel immunotherapy target for triple negative breast cancer. Breast Cancer Res Treat. 2012;133(2):799–804.
12. Rizk NP, Servais EL, Tang LH, Sima CS, Gerdes H, Fleisher M, Rusch VW, Adusumilli PS. Tissue and serum mesothelin are potential markers of neoplastic progression in Barrett's associated esophageal adenocarcinoma. Cancer Epidemiol Biomark Prev. 2012;21(3):482–6.
13. Morello A, Sadelain M, Adusumilli PS. Mesothelin-targeted CARs: driving T cells to solid tumors. Cancer Discov. 2016;6(2):133–46.
14. Pastan I, Hassan R. Discovery of mesothelin and exploiting it as a target for immunotherapy. Cancer Res. 2014;74(11):2907–12.
15. Nichetti F, Marra A, Corti F, Guidi A, Raimondi A, Prinzi N, de Braud F, Pusceddu S. The Role of Mesothelin as a Diagnostic and Therapeutic Target in Pancreatic Ductal Adenocarcinoma: A Comprehensive Review. Target Oncol. 2018 Jun;13(3):333-351.

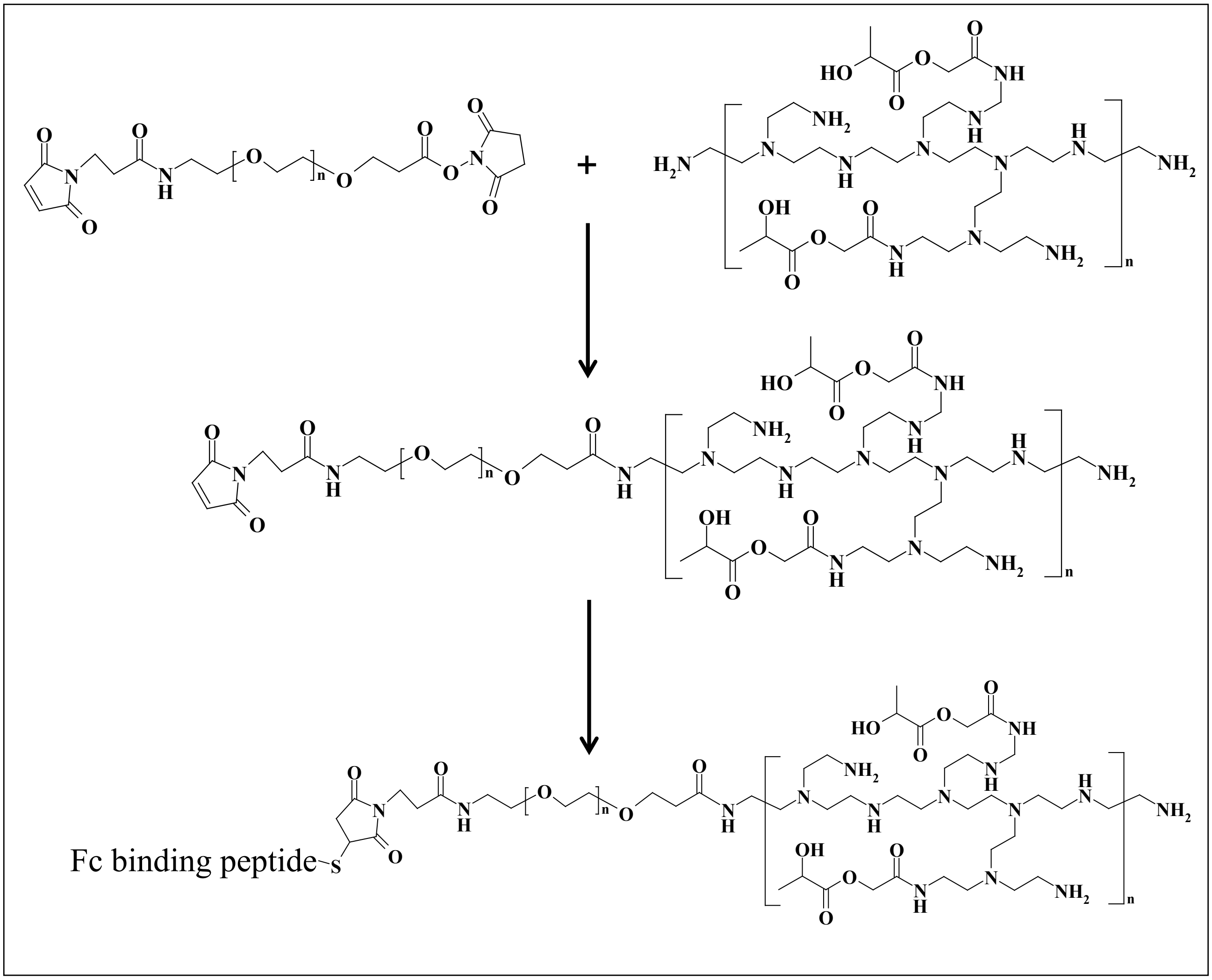

Figure S1. Synthesis of FcBP-LGA-PEI polymer and nanoparticle (NP) formation with antibody (Ab) and nucleic acids. Conjugation of FcBP with LGA-PEI polymer for binding full Ab for the targeting delivery. PEG (Mal-PEG-NHS) was covalently linked to LGA-PEI. FcBP (CGGGGDCAWHLGELVWCTGGGGC) was covalently linked to the Mal-PEG-PEI-LGA though a click reaction.

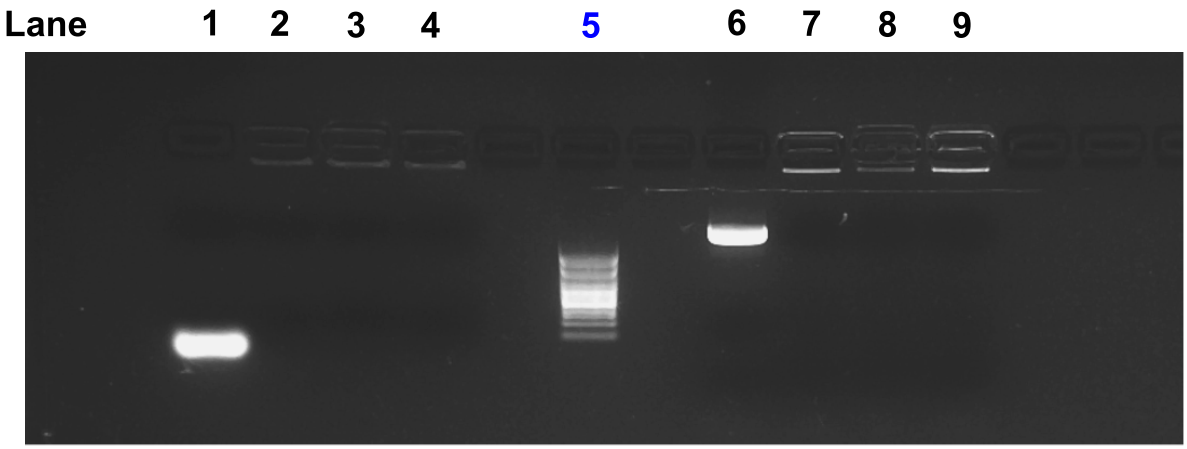

1. **miR-198 mimic**
2. **LGA-PEI/miR-198 mimic NPs**
3. **MSLN scFv-PEI/miR-198 mimic NPs**
4. **Anti-EGFR Ab/FcBP-LGA-PEI/miR-198 mimic NPs**
5. **Molecular markers (ladder)**
6. **Plasmid-GFP (4.6 kb)**
7. **LGA-PEI/plasmid-GFP NPs**
8. **MSLN scFv-LGA-PEI/plasmid-GFP NPs**
9. **Anti-EGFR Ab/FcBP-LGA-PEI/plasmid-GFP NPs**

Figure S2. Gel retardation assay to determine the nucleic acid load efficiency of our polymers (LGA-PEI, MSLN scFv-LGA-PEI and anti-EGFR Ab/FcBP-LGA-PEI). All polymers are able to load different forms of nucleic acids (miR-198 mimics or GFP plasmid DNA) and form nanoparticles. All loaded nucleic acids with all polymer-based nanoparticles retained inside the sample loading well of the gel without migration in the electrophoretic field, indicating a high load efficiency of nucleic acids and nanoparticle formation of our polymers. The RNA nanoparticles were prepared with 1 μg miR-198 mimics and 1.5 μg LGA-PEI, MSLN scFv-LGA-PEI or anti-EGFR Ab/FcBP-LGA-PEI, respectively, in 10 μl water; while DNA nanoparticles were prepared with 1 μg GFP-containing plasmid DNA (4.6kb) and 1.5 μg LGA-PEI, MSLN scFv-LGA-PEI or Anti-EGFRAb/FcBP-LGA-PEI, respectively, in 10 μl water. The particles were kept at room temperature for 30 min before applied to 1% agarose gel retardation assay. For the negative control, miR-198 mimics (1 μg) or GFP-containing plasmid DNA (1 μg) was included in the experiment. For each nanoparticle or control, the whole 10 μl sample was added to the sample well of the gel. The gel electrophoresis ran at 150 V for 20 min and gel retardation was visualized under UV light.

Table S3. Nanoparticle sizes for different ratios of MSLN scFv-LGA-PEI and LGA-PEI (total 5 μg) and the same amount of the plasmid DNA (2 μg) (See discussion below**)

% MSLN scFv-LGA-PEI	% LGA-PEI	Plasmid DNA (μg)	Diameter (size) (nm)	SD	PDI
100	0	2	112.6	35.9	0.223±0.008
50	50	2	133.2	65	0.221±0.007
30	70	2	146.2	57.4	0.194±0.010
20	80	2	153.9	57.1	0.188±0.008
10	90	2	136.1	53.1	0.208±0.014
5	95	2	147.5	54.6	0.169±0.015
1	99	2	120.1	46.6	0.156±0.006
0	100	2	153.4	61.2	0.186±0.012

Table S4. Nanoparticle sizes for different ratios of MSLN scFv-LGA-PEI and LGA-PEI (total 1.5 μg) and the same amount of miR-198 mimics (1 μg) (See discussion below**)

% MSLN scFv-LGA-PEI	% LGA-PEI	miR-198 mimics (μg)	Water (μl)	Dilution in water (ml)	Diameter (size) (nm)	SD	PDI
100 (1.5 μg)	0	1	25	1	126.2	27.6	0.326±0.058
50 (0.75 μg)	50 (0.75 μg)	1	25	1	123.9	24.5	0.227±0.055
30 (0.5 μg)	70 (1 μg)	1	25	1	136.9	31.9	0.316±0.047
20 (0.3 μg)	80 (1.2 μg)	1	25	1	113.0	23.9	0.234±0.050
10 (0.15 μg)	90 (1.35 μg)	1	25	1	147.9	22.6	0.364±0.078
5 (0.075 μg)	95 (1.425 μg)	1	25	1	235.9	61.9	0.077±0.041
1 (0.015 μg)	99 (1.5 μg)	1	25	1	226.8	60	0.200±0.047
0	100 (1.5 μg)	1	25	1	331.9	63.5	0.202±0.051

Table S5. Nanoparticle sizes for different ratios of anti-EGFR Ab/FcPB-LGA-PEI and LGA-PEI (total 5 μg) and the same amount of plasmid DNA (2 μg) (See discussion below**)

% Anti-EGFR-FcBP-LGA-PEI	% LGA-PEI	Plasmid DNA (μg)	Water (μl)	Dilution in water (ml) after 30 min	Diameter (size) (nm)	SD	PDI
100 (5 μg)	0	2	50	0.95	113.3	47.5	0.221±0.016
50 (2.5 μg)	50 (2.5 μg)	2	50	0.95	121.4	44.8	0.150±0.008
30 (1.5 μg)	70 (3.5 μg)	2	50	0.95	106.9	34.6	0.156±0.015
20 (1 μg)	80 (4 μg)	2	50	0.95	123.1	46.2	0.152±0.014
10 (0.5 μg)	90 (4.5 μg)	2	50	0.95	123.2	49	0.137±0.006
5 (0.25 μg)	95 (4.75 μg)	2	50	0.95	121.3	44.5	0.147±0.026
1 (0.05 μg)	99 (4.95 μg)	2	50	0.95	123.7	55.3	0.169±0.009
0	100 (5 μg)	2	50	0.95	119.1	44.4	0.153±0.021

****Discussion** for Table S3, Table S4, and Table S5.

The average diameter (size) of nanoparticles shown in the Table S3, Table S4 and Table S5 was obtained from multiple measurements of the sample by Malvern Zetasizer. Mean size, also called Z-average (diameter in nm), is automatically calculated from the intensity weighted distribution from Malvern Zetasizer. Standard deviation (SD) represents the intensity distribution (68.2% of the total distribution lies within ± 1σ). The size distributions of our nanoparticles shown in the tables above are of high quality and scientifically acceptable based on their polydispersity indexes (PDI), which is an important parameter used to define the size range of the particles. PDI serves as a heterogeneity index, and it is a number calculated from a two-parameter fit to the correlation data (the cumulants analysis). In drug delivery applications of nanoparticles, a PDI of 0.3 and below is considered to be acceptable and indicates a homogenous population of delivery nanoparticles (references 1-4). PDIs in our experiments range from 0.1 to 0.3 with very small SDs.

Thus, we have produced high quality nanoparticles from our polymers (LGA-PEI, MSLN scFv-LGA-PEI, anti-EGFR Ab/FcBP-LGA-PEI) with different forms of nucleic acids.

References

1. Danaei, M.; Dehghankhold, M.; Ataei, S.; Hasanzadeh Davarani, F.; Javanmard, R.; Dokhani, A,; Khorasani, S.; Mozafari, M.R. Impact of particle size and polydispersity index on the clinical applications of lipidic nanocarrier systems. *Pharmaceutics.* **2018,** *10,* 57.
2. Badran, M. Formulation and in vitro evaluation of flufenamic acid loaded deformable liposome for improved skin delivery. *Digest J. Nanomater. Biostruct.* **2014,** *9,* 83–91.
3. Chen, M.; Liu, X.; Fahr, A. Skin penetration and deposition of carboxyfluorescein and temoporfin from different lipid vesicular systems: In vitro study with finite and infinite dosage application. Int. J. Pharm. **2011,** *408,* 223–234.
4. Putri, D.C.; Dwiastuti, R.; Marchaban, M.; Nugroho, A.K. Optimization of mixing temperature and sonication duration in liposome preparation. *J. Pharm. Sci. Commun.* **2017,** *14,* 79–85.

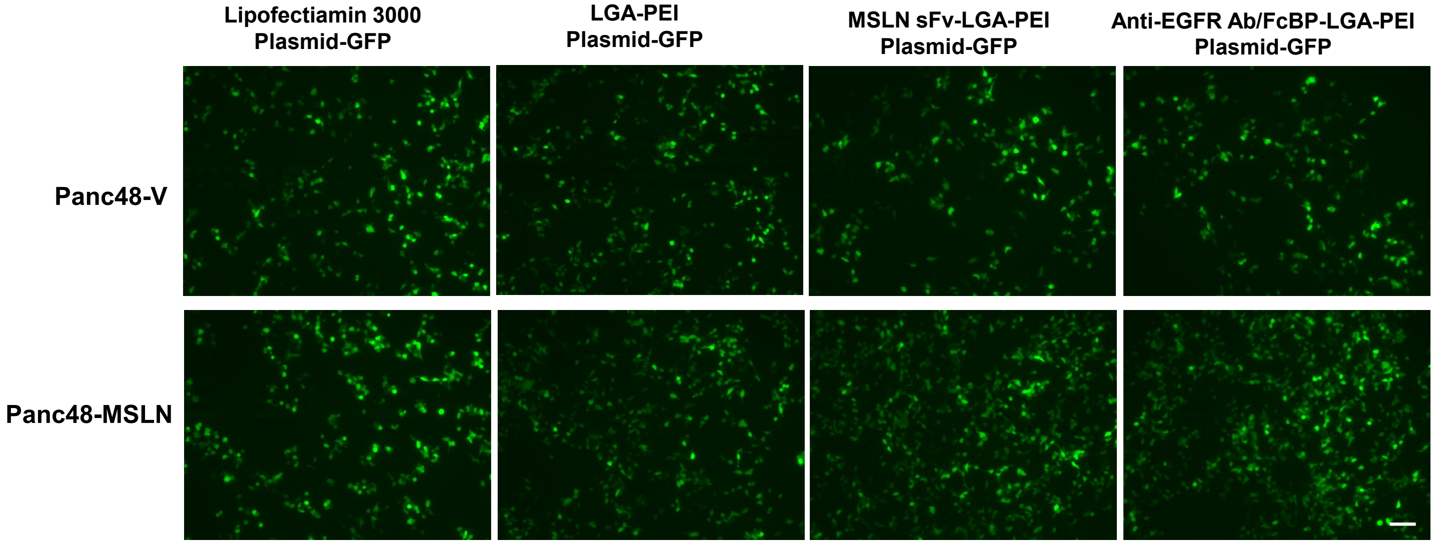

Figure S3. Transfection efficiency of green fluorescence protein (GFP) gene mediated by different polymers (LGA-PEI, MSLN scFv-LGA-PEI and anti-EGFR Ab/FcBP-LGA-PEI) and commercial lipofectamine 3000 in MSLN overexpression and vector control cells *in vitro*. MSLN overexpressed PDAC cells (Panc48-MSLN) or vector control cells (Panc48-V) (2.5×10^5) were seeded in 12-well plates and cultured with DMEM medium (10% FBS) one day prior to cell transfection. With overnight culture in cell incubator (5% CO_2,37°C), PDAC48 cells reached 70-90% confluency, and GFP containing plasmid DNA (1 μg)-lipid complexes were produced and added directly to cells in culture medium according to the protocol of Lipofectamine 3000 Reagent (Thermo Fisher Scientific, Cat# L3000015). For NP-mediated transfection, different types of nanoparticles (NPs) were prepared by GFP plasmid DNA (1 μg) mixed with LGA-PEI, MSLN scFv-LGA-PEI or anti-EGFR Ab/FcBP-LGA-PEI (3.5:1, polymer/DNA ratio) were added into the cell culture of PDAC48 cells. After 24 h incubation, gene expression in transfected cells was measured by observing GFP-positive cells under a fluorescent microscope. As a control, commercial lipofectamine 3000 was used to deliver GFP plasmid DNA into Panc48-MSLN and Panc48-V cells; however, its transfection efficiency had no difference between two cell types. The transfection efficiency of LGA-PEI/GFP-plasmid DNA NPs was similar in two cell types, indicating no targeted delivery. By contrast, MSLN scFv-LGA-PEI/GFP-plasmid DNA NPs or anti-EGFR Ab/FcBP-LGA-PEI/GFP-plasmid DNA NPs had a higher transfection efficiency in Panc48-MSLN cells than that in Panc48-V cells, indicating targeted delivery of GFP-plasmid DNA. Lipofectamine 3000 cannot be *in vivo* due to high toxicity, while our polymer-based NP delivery systems can be used *in vivo* and have potential clinical applications. We previously demonstrated that MSLN could enhance EGFR expression in PC cell lines (reference 1). Scale bar (50 μm). Pancreatic cancer cell line Panc48 cells were infected with lentivirus to express an empty vector or human MSLN cDNA (NM_005823) vector by using the pLenti6.3/V5-TOPO TA Cloning Kit (K531520; Invitrogen). Stable vector or MSLN-overexpressing cell lines were selected by using 5 μg/ml blasticidin. Overexpression of MSLN of Panc48 cells transfected with MSLN cDNA (Panc48 MSLN) was confirmed by Western blot analysis; while Panc48 transfected with empty vector (Panc48-V) had very low MSLN expression.

Reference

1. Poteet E, Liu D, Liang Z, Van Buren G, Chen C, Yao Q. Mesothelin and TGF-α predict pancreatic cancer cell sensitivity to EGFR inhibitors and effective combination treatment with trametinib. *PLoS ONE* **2019**, *14*, e0213294.
